# CT and MR imaging prior to transcatheter aortic valve implantation: standardisation of scanning protocols, measurements and reporting—a consensus document by the European Society of Cardiovascular Radiology (ESCR)

**DOI:** 10.1007/s00330-019-06357-8

**Published:** 2019-09-05

**Authors:** Marco Francone, Ricardo P. J. Budde, Jens Bremerich, Jean Nicolas Dacher, Christian Loewe, Florian Wolf, Luigi Natale, Gianluca Pontone, Alban Redheuil, Rozemarijn Vliegenthart, Kostantin Nikolaou, Matthias Gutberlet, Rodrigo Salgado

**Affiliations:** 1 Department of Radiological, Oncological and Pathological Sciences, Sapienza University, Policlinico Umberto I, V.le Regina Elena 324, 00161 Rome, Italy; 2 Department of Radiology & Nuclear Medicine, Erasmus MC, Rotterdam, The Netherlands; 3 Department of Radiology, University of Basel Hospital, Basel, Switzerland; 4 Department of Radiology, Normandie University, UNIROUEN, INSERM U1096 – Rouen University Hospital, F 76000 Rouen, France; 5 Division of Cardiovascular and Interventional Radiology, Department of Biomedical Imaging and Image-Guided Therapy, Medical University of Vienna, Vienna, Austria; 6 Department of Radiological Sciences - Institute of Radiology, Catholic University of Rome, “A. Gemelli” University Hospital, Rome, Italy; 7 Centro Cardiologico Monzino, IRCCS, Milan, Italy; 8 Institute of Cardiometabolism and Nutrition (ICAN), Paris, France; 9 Department of Cardiovascular and Thoracic, Imaging and Interventional Radiology, Institute of Cardiology, APHP, Pitié-Salpêtrière University Hospital, Paris, France; 10 Laboratoire d’Imagerie Biomédicale, Sorbonne Universités, UPMC Univ Paris 06, INSERM 1146, CNRS 7371, Paris, France; 11 Department of Radiology, University of Groningen, University Medical Center Groningen, Groningen, Netherlands; 12 Department of Diagnostic and Interventional Radiology, University of Tuebingen, Tübingen, Germany; 13 Diagnostic and Interventional Radiology, University of Leipzig-Heart Center, Leipzig, Germany; 14 Department of Radiology, Antwerp University Hospital, Antwerp, Belgium; 15 Department of Radiology, Holy Heart Hospital, Lier, Belgium

**Keywords:** Transcatheter aortic valve replacement, Aortic valve stenosis, Consensus, Multidetector computed tomography, Magnetic resonance imaging

## Abstract

**Abstract:**

Transcatheter aortic valve replacement (TAVR) is a minimally invasive alternative to conventional aortic valve replacement in symptomatic patients with severe aortic stenosis and contraindications to surgery. The procedure has shown to improve patient’s quality of life and prolong short- and mid-term survival in high-risk individuals, becoming a widely accepted therapeutic option which has been integrated into current clinical guidelines for the management of valvular heart disease. Nevertheless, not every patient at high-risk for surgery is a good candidate for TAVR. Besides clinical selection, which is usually established by the Heart Team, certain technical and anatomic criteria must be met as, unlike in surgical valve replacement, annular sizing is not performed under direct surgical evaluation but on the basis of non-invasive imaging findings. Present consensus document was outlined by a working group of researchers from the European Society of Cardiovascular Radiology (ESCR) and aims to provide guidance on the utilisation of CT and MR imaging prior to TAVR. Particular relevance is given to the technical requirements and standardisation of the scanning protocols which have to be tailored to the remarkable variability of the scanners currently utilised in clinical practice; recommendations regarding all required pre-procedural measurements and medical reporting standardisation have been also outlined, in order to ensure quality and consistency of reported data and terminology.

**Key Points:**

*• To provide a reference document for CT and MR acquisition techniques, taking into account the significant technological variation of available scanners.*

*• To review all relevant measurements that are required and define a step-by-step guided approach for the measurements of different structures implicated in the procedure.*

*• To propose a CT/MR reporting template to assist in consistent communication between various sites and specialists involved in the procedural planning.*

**Electronic supplementary material:**

The online version of this article (10.1007/s00330-019-06357-8) contains supplementary material, which is available to authorized users.

## Introduction

Elective surgical aortic valve replacement (AVR) is considered the most effective treatment for advanced aortic valve stenosis (AS), significantly improving symptoms and survival in comparison with individuals who refused or could not undergo an invasive surgical procedure. Despite a reported mortality rate of 50% in the first 2 years for untreated patients, 30–40% of individuals could not receive curative treatment, deemed to be ineligible to surgery because of the high peri-operative risk [1, 2].

In response, new procedural options have emerged, based on the development of transcatheter therapies with specific aortic valve prostheses that can be transported to the aortic root using a non-surgical endovascular, transaortic or transapical approach. Once in place, these bioprosthetic valves or transcatheter heart valves (THVs) functionally replace the native valve by displacing it to the aortic root wall during deployment. This procedure is named transcatheter aortic valve replacement or implantation (TAVR or TAVI) or percutaneous aortic valve replacement (PAVR) and was introduced in 2002 [3]. An illustrative guide explaining the procedure for a self-expandable valve is displayed in Supplementary Material 1.


ESM 4(MP4 24834 kb)


Nevertheless, not every patient who refused or at high-risk for surgery is a good candidate for TAVI. Besides clinical selection, certain technical and anatomic criteria must be met as, unlike in surgical valve replacement, annular sizing is not performed under direct surgical inspection but on the basis of non-invasive imaging findings.

It has been estimated that annually about 27,000 individuals with AS potentially fulfil eligibility criteria for TAVI in Europe and North America, with obvious economic, clinical and social implications emphasising the importance of an adequate candidate selection [4, 5].

The present consensus document was outlined by a working group of radiologists and researchers from the European Society of Cardiovascular Radiology (ESCR) and aims to provide guidance on the execution and reporting of CT and MR imaging prior to TAVI. Particular relevance will be given to the technical requirements and standardisation of the scanning protocols which have to be tailored to the considerable variation of the scanner technology currently utilised in clinical practice; recommendations regarding standardised measurements and medical reporting will also be outlined, in order to ensure quality and consistency of reported data and terminology.

## Heart valve team

### Consensus statement

- *The Heart Valve Team supervises and discusses all aspect of the TAVI selection process. A radiologist forms an integral part of this team.*

The workup of a patient candidate for TAVI is a complex and multifactorial process. Beyond patient selection and evaluation, many factors contribute to the final success of the entire procedure, such as team training and experience, procedural performance, complication management, and post-procedural follow-up.

Therefore, it is recommended that all centres performing TAVI procedures have extensive experience as a heart valve centre including the availability of a dedicated Heart Valve Team composed of experts in their respective field. Their task is to supervise every aspect of the decision-making progress and to assess individual patient risk for the different available treatment options, with a shared decision-making approach for the optimal therapeutic option. As such, the final decision regarding a TAVI procedure must rely on the combination of all available clinical data and imaging data from different modalities.

As non-invasive CT and MR imaging delivers essential information necessary for proper patient selection and procedural success, an experienced radiologist must form an integral part of the core team, combining clinical and technical knowledge and discussing all relevant CT (or MR) imaging findings with the rest of the team [6].

## Indications for TAVI

### Consensus statement

- *TAVI is primarily targeted at high-risk non-surgical patients with severe AS. Recent trials results indicate a potential expansion to intermediate-risk patients, as further evidence is gathered.*

In the last decade, TAVI has emerged as a transformational technology providing new therapeutic options for selected adult patients with severe AS. As intended, TAVI is approved by different societies to be used in patients with severe symptomatic AS at high prohibitive surgical risk and a life expectancy of more than 1 year [7–10] (Table 1). The EuroSCORE II [11] and/or the Society of Thoracic Surgeon (STS) risk score [12] is employed to predict the procedural risk of surgical valve replacement. High-risk surgery corresponds to a EuroSCORE II > 15–20% or STS score > 8–10%.

**Table 1:** Currently accepted clinical indications to TAVI summarised from the ESC/EACTS and AHA/ACC guidelines and from recently updated ACC/AHA expert consensus decision pathway [7, 9]

	ACC/AHA*	ESC/EACTS^
Approach to care and clinical decision-making:	To be established by a shared decision of local heart team	To be made by a “heart team” with specific expertise in VHD
Indications to the procedure:	Recommended in patients with indication to intervention for AS combined with a prohibitive surgical risk and a predicted post-procedural survival > 12 months	Indicated in patients with severe AS and contraindication to surgery, with an estimated life expectancy > 1 year and an expected improvement of QoL by TAVI
General contraindications:	Overall procedural risks and contraindications based on scores evaluating patient’s frailty and disability plus cognitive and physical *function*	General absolute contraindications include the absence of a local “heart team” and/or an on-site cardiac surgery facility
Importance of comorbidities:	Procedure considered futile if life expectancy < 1 year and/or survival with benefit < 25% at 2 years (i.e. lack of improvement in NYHA or CCS functional classes, quality of life or life expectancy*)*	Contraindicated In presence of extra-aortic valvular disease that can be treated only by surgery and/or in presence of an estimated life expectancy < 1 year and/or unlikely post-procedural improvement of QoL
Anatomic contraindications:	Non-specified (considered part of the clinical decision-making process performed by local heart team)	Inadequate annulus sizing (i.e. < 18 mm and a 29 mm) Intracavitary thrombus, endocarditis, risk of coronary ostium obstruction and ascending aorta/arch unstable atheromasia Inadequate vascular access

*ACC/AHA: American College of Cardiology/American Heart Association

^ESC/EACTS: European Society of Cardiology/European Association of Cardio-Thoracic Surgery

However, results of recent trials indicate a potential expansion of TAVI indications to patients with an intermediate surgical risk, applicable to both self-expandable and balloon-expandable valves [8, 10, 13, 14]. This is also commented on in the latest European Society of Cardiology guidelines [7, 15]. This reflects the accumulation of data regarding its efficacy and non-inferiority compared with a surgical approach in this patient category. Nevertheless, the final decision regarding the therapeutic procedure is not simply based on a risk score but is the end result of the deliberations by the Heart Valve Team regarding the risk and benefits of all possible interventions, especially in the intermediate-risk group. Also, concerns remain regarding the long-term durability of THV, an important point to consider when applying this technique in a younger, lower risk population.

Other indications, including the use in low-risk patients and the application of THV to treat bicuspid aortic valve disease and aortic regurgitation, are currently under investigation. Nevertheless, lack of high-quality data regarding the mentioned long-term performance of THV and together with other on-going issues like prevalence of post-procedural paravalvular leakage and conduction disturbances with subsequent need for pacemaker implantation remain important obstacles. Therefore, a TAVI procedure in these conditions is not recommended in routine clinical practice.

A relatively new application for TAVI is the treatment of a failing surgical biological aortic valve prosthesis: the so-called valve-in-valve procedure. This option is particularly beneficial for high-risk individuals who underwent previous valvular surgery. This procedure consists of placing a TAVI prosthesis within a degenerated surgical bioprosthetic valve [16].

## Diagnosis of severe aortic valve stenosis

### Consensus statement

- *The diagnosis and grading of severe aortic valve stenosis relies on the patients’ symptoms and imaging data regarding aortic valve anatomy & hemodynamics.*
- *This imaging data is commonly acquired using Doppler echocardiography.*
- *Quantification of the aortic valve calcification load based on CT for diagnostic purposes is only necessary in selected patients with a discordant result on Doppler echocardiography.*
- *MRI can be used for quantification of the aortic valve opening area and transvalvular velocities using planimetry and phase contrast imaging with simultaneous LV ejection fraction calculation*

Although, as previously discussed, indications for TAVI are expanding; the main indication for TAVI remains severe symptomatic aortic stenosis in high-surgical-risk patients.

Transthoracic echocardiography is the primary imaging tool to diagnose AS, to confirm its presence, determine its severity and deliver both anatomical and functional information. Quantitative and qualitative data are provided using Doppler techniques, resulting in an assessment of AS severity.

In the case of normal transaortic volume flow rate, the best characterisation of hemodynamic severity is achieved by the assessment of the transaortic maximum velocity, mean pressure gradient and aortic valve opening area (AVA). The maximum transaortic velocity is measured using continuous wave Doppler and the mean pressure gradient calculated based on a tracing of the Doppler signal. AVA is not measured directly but calculated using the continuity equation. In severe high-gradient AS, the maximal aortic velocity is 4.0 m/s or higher and the mean transaortic gradient is ≥ 40 mmHg. The aortic valve area is ≤ 1.0 cm^2^, but it may be larger under certain conditions.

However, two different categories of severe AS may exist where transaortic volume flow rate is low. Left ventricle systolic dysfunction with low left ventricle ejection fraction (LVEF) defines a low-flow/low-gradient severe AS subgroup. Furthermore, the presence of a small hypertrophied left ventricle with a low stroke volume and normal LVEF points to paradoxical low-flow severe AS. In the case of reduced LVEF, dobutamine stress echocardiography can be used to assess whether LVEF can increase with a resultant increased transaortic flow and increased aortic velocity to more than the 4.0 m/s threshold. Overall, the final diagnosis of severe AS in these conditions, and therefore the potential need for TAVI, may be difficult to establish using only echocardiography.

While exact quantification of aortic valve calcification load is not routinely done in most centres, it may be considered when Doppler echocardiography results are discordant with the presumed diagnosis of AS. In such patients, quantification of aortic valve calcification using the Agatston method, based on a non-contrast CT, can be problem-solving, as AS severity is associated with the load of valve calcification and provides additional diagnostic value beyond clinical and Doppler echocardiographic assessment [17, 18].

Different cut-off Agatston score values that make severe AS *likely* have been proposed as ≥ 2000 for men and ≥ 1200 for women, as women have more severe AS for the same calcium load compared with men [19, 20]. Scores of ≥ 3000 for men and ≥ 1600 for women are considered to make AS *very likely* [21]*.* The latter thresholds are probably based on the study by Clavel et al, in which these values give an approximately 95% positive predictive value for severe AS [18]. The same CT protocol is commonly similar to the one used for Calcium scoring of the coronary arteries that by convention is performed at 120 KV and with 3-mm thickness axial reconstructions. Only the calcifications on the aortic valve leaflets are to be included in the calculation [17]. Although direct planimetry of the AVA on systolic CTA correlates with Doppler-derived AVA, there is currently no direct role in the diagnostic pathway for severe AS [22].

Alternatively, MRI can be used for assessment of aortic valve stenosis in the following ways: direct aortic valve opening area measurement using planimetry using in-plane systolic images; phase contrast velocity mapping for calculation of pressure gradients over the valve and left ventricular function from routine SSFP cine sequences [23]. Multiple double-oblique parallel slices in plane with the valve should be acquired to select the optimal phase for AVA measurement (phase with maximum opening of the valve, measured at the smallest orifice). Phase contrast flow calculations can result in an underestimation of the flow compared with echocardiography due to inherent differences between the two techniques and can also be influenced by turbulent flow. Furthermore, planimetry can be difficult in heavily calcified valves.

## Pre-procedural comorbidities and incidental findings

### Consensus statement

- *CT must not be routinely used for pre-procedural evaluation of coronary artery disease. However, as technology evolves, it can be used for this indication on a case-by-case basis and according to local expertise and available equipment and mainly to exclude significant coronary stenosis.*
- *Repercussions of incidental findings, including the presence of malignancy, must be evaluated by the Heart Valve Team on a case-by-case basis with regards to their influence on procedural success and prognosis. Every finding that can influence the procedure and its outcome must be reported.*

Outside the diagnosis of severe AS, the assessment of comorbidities is of pivotal importance and needs a careful case-by-case analysis. Given the advanced age, frail condition and varying pre-existing conditions of TAVI candidates, a careful multidisciplinary analysis is needed not only to assure procedural eligibility but also to assess the likelihood of post-procedural functional improvement and enhanced quality of life [24]. In this document, we will focus on the contributions of the radiologist.

Coronary artery disease is common (40–75%) in patients undergoing TAVI and, in the absence of up-to-date information regarding the status of the coronary arteries (no longer than 3 months old), further investigation is needed [25]. In general, coronary artery evaluation is commonly performed using a classic invasive angiography. It is, currently, not recommended to routinely use CT for the pre-procedural evaluation of the coronary arteries as the investigated population is less suitable (prevalence of extensive coronary artery calcifications and cardiac arrhythmia) for coronary CT scanning. Nevertheless, given advances in CT technology with increasingly reliable image quality under a wider spectrum of conditions, CT may be considered on a case-by-case basis to exclude obstructive coronary artery disease, based on the locally available equipment and expertise. An additional benefit of CT is that it can allow concomitant evaluation of the coronary artery status together with the other necessary pre-procedural measurements in a single CT examination and intravenous contrast administration, as such limiting potential contrast-induced nephrotoxicity.

Evaluation of non-vascular findings is an integral part of the radiology report, and clinically significant incidental findings have been reported in up to 25% of TAVI candidates [26]. Given the dismal prognosis of untreated severe AS, important incidental findings like unexpected malignancy must be evaluated on a case-by-case basis by the Heart Valve Team, consulting other relevant physicians and all available clinical data. Incidental findings with immediate impact on the procedure, e.g. obstruction along the possible access routes, have to be highlighted in the radiology report as they may influence procedure eligibility.

## Types of valvular devices and access sites

### Consensus statement

- *Balloon-expandable and self-expandable valves have different physical properties and possible access strategies. Therefore, sizing algorithms are not simply interchangeable and do not follow specific guidelines.*
- *The choice for a valve type mainly depends on the experience of the Heart Valve Team with a particular valve, and on the available access routes.*

All current clinically implemented THVs fall into two categories: balloon-expandable (BE) or self-expandable (SE) valves [19]. A BE valve will expand using the radial strength of the accompanying balloon and commonly force its circular design on the oval-shaped annular morphology. Conversely, a SE valve will deploy until it encounters the resistance of the annular wall, conforming itself to the mostly oval-shaped anatomy of the aortic annulus. This difference in physical properties and consequently post-procedural morphology of the THV implies that sizing algorithms are not interchangeable between balloon- and self-expandable valves.

**Fig. 1:**
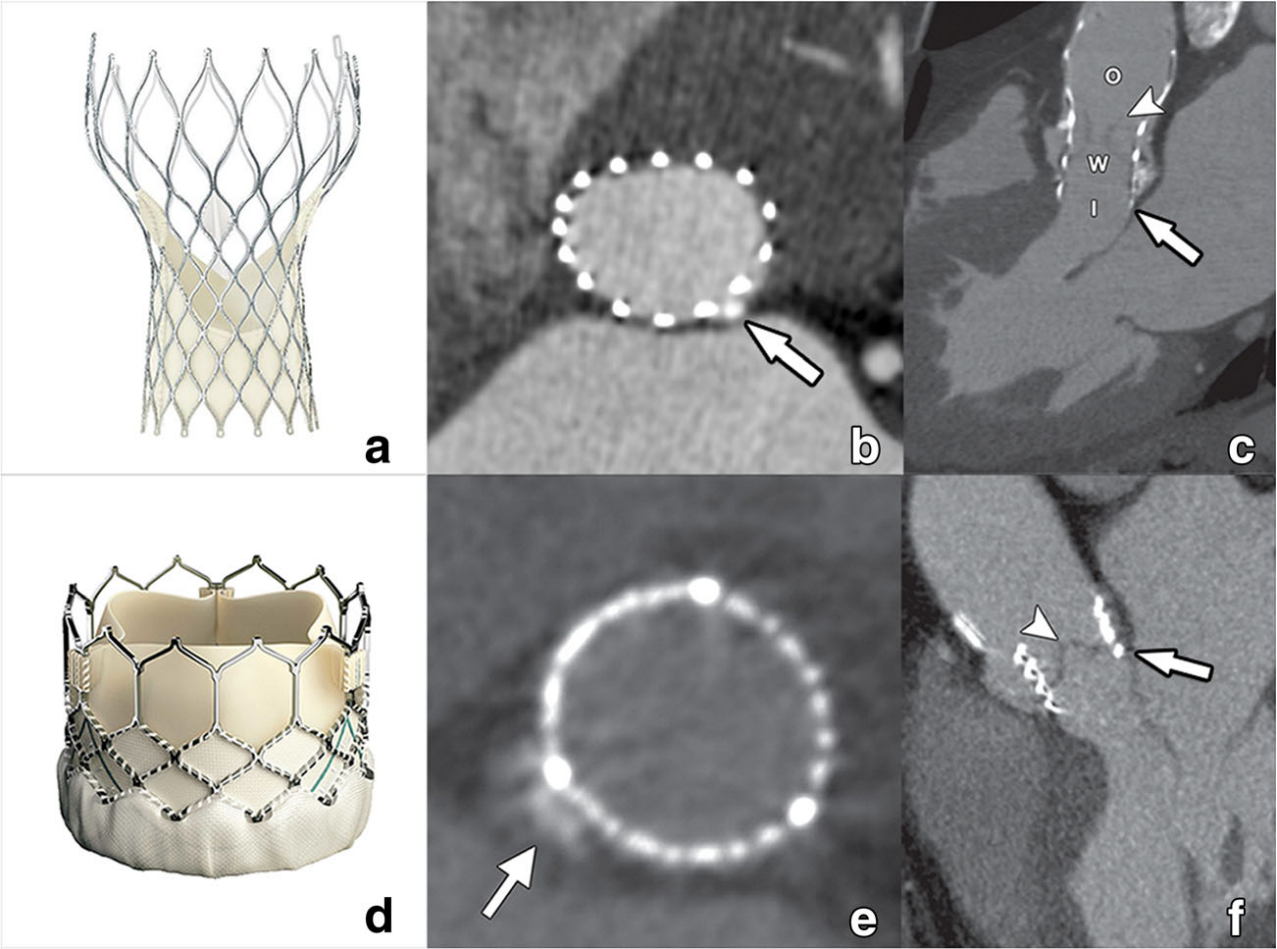
CT images of a Medtronic self-expandable Corevalve (**a**) and Edwards Lifesciences balloon-expandable Sapien valve (**d**). After deployment, a self-expandable valve will conform itself to the normal annular contour, acquiring an oval cross-sectional morphology (**b**). Compared with a balloon-expandable valve, the self-expandable Corevalve is larger in size and contains an inflow (I), waist (w), and outflow (O) functional part (**c**). This outflow part is by design intended to extend into the ascending aorta, covering but not obstructing the coronary ostia. The balloon-expandable Sapien valve (**d**) is shorter, with a mostly circular cross-sectional contour after deployment (**e**) as it forces this circular morphology on the annulus through the radial forces of the expanding balloon. In contrast to the self-expandable Corevalve, it remains within the aortic sinus (**f**). Within both THVs, the pericardial leaflets can be appreciated as fine hypodense linear structures (arrowheads in **c**, **f**), its visibility dependent on image quality. Small interposing calcifications (arrows in **b**, **e**) have in these examples only a minimal effect on THV expansion. Reused from reference [40], with permission

The most commonly used THVs are the BE SAPIEN range (currently SAPIEN S3) from Edwards LifeSciences (Irvine, Calif), and the SE Corevalve from Medtronic (Minneapolis, Minn), now replaced by the newer Evolut platform (Fig. 1). A detailed overview of the properties of these most commonly used THV is given in Table 2. Both systems are approved in Europe and in the USA for use in patients with severe symptomatic AS who are considered to be at high surgical risk or who declined for surgery owing to excessive risk.

**Table 2:** Physical properties of most commonly used THV and sizing range (chapter 6)

	SAPIEN 3	Evolut PRO/R
Manufacturer	Edwards Lifesciences	Medtronic
Available sizes (mm)	20	23
	23	26
	26	29
	29	34
Annular range TEE (mm)	16–28	17/18–30 (17 for valve-in-valve only)
Deployment	Balloon-expandable	Self-expandable
Frame	Cobalt-chromium	Nitinol
Frame height (mm)	18–22.5	45 (46 mm for 34-mm valve)
Pericardial leaflets	Bovine	Porcine
Valve function	Intra-annular	Supra-annular
Repositionable	No	Yes
Ascending aorta fixation	No	No
Access routes	Transfemoral	Transfemoral
	Transapical	Transaxillary
	Transaortic	Transaortic
Transfemoral delivery sheath size	14F (16F for 29 mm valve)	16F

These devices cover a combined aortic annular diameter range of 16–30 mm, thereby allowing application in the vast majority of patients. Also, they have different physical properties, potential access routes for delivery and choice of delivery systems. All these factors may come to play a role in the final choice of valve and delivery route.

There are currently no guidelines regarding the choice between self-expandable and balloon-expandable valves. In general, many centres acquire experience using mainly one type of valve in order to achieve the highest procedural success. Selection of a valve type mainly depends on the range of valve sizes available, the dimensions of the delivery device versus the native vessels and the possible access routes.

There are some indications that a SE valve may be preferable in an extremely oval-shaped annulus or a low implantation of coronary ostia. Conversely, a BE device can be considered when patients have a dilated ascending aorta (> 43 mm) or severely angulated aorta (aortoventricular angle > 70°) [27]. Despite wide diffusion and favourable outcomes using the first generation of THV, some limitations remain. These include, among others, the use of larger delivery systems increasing the risk for haemorrhagic and other vascular complications during endovascular transportation and a percentage of patients with annular dimensions outside the applicable range of these devices. Other complications such as post-procedural paravalvular regurgitation and conduction disturbances are multifactorial, but seem in part also linked to the type of THV used (e.g. more conduction disturbances in SE valves) [28, 29]. Additionally, none of the first-generation devices could be repositioned, requiring implantation of a second prosthesis or referral for surgery in case of unsatisfactory positioning [21].

Therefore, various new THVs are being developed by different biomedical companies to overcome the drawbacks of the first-generation TAVI devices and are in varying states of large-scale clinical testing and official approval [30]. As an example, while both SE and BE THV valves can be implanted using an endovascular approach, only the SAPIEN valve was the first to have the option of a transapical approach, bypassing heavily calcified or tortuous/stenotic native arteries. Therefore, recently newer SE systems have been introduced in the market to take specific advantage of a transapical approach including the Engager, JenaValve and Acurate valves [31]. Summarising physical properties, strengths and weaknesses of these new-generation devices would go beyond the scope of this document. A brief overview of last generations’ devices is displayed in Table 3**.**

**Table 3:**
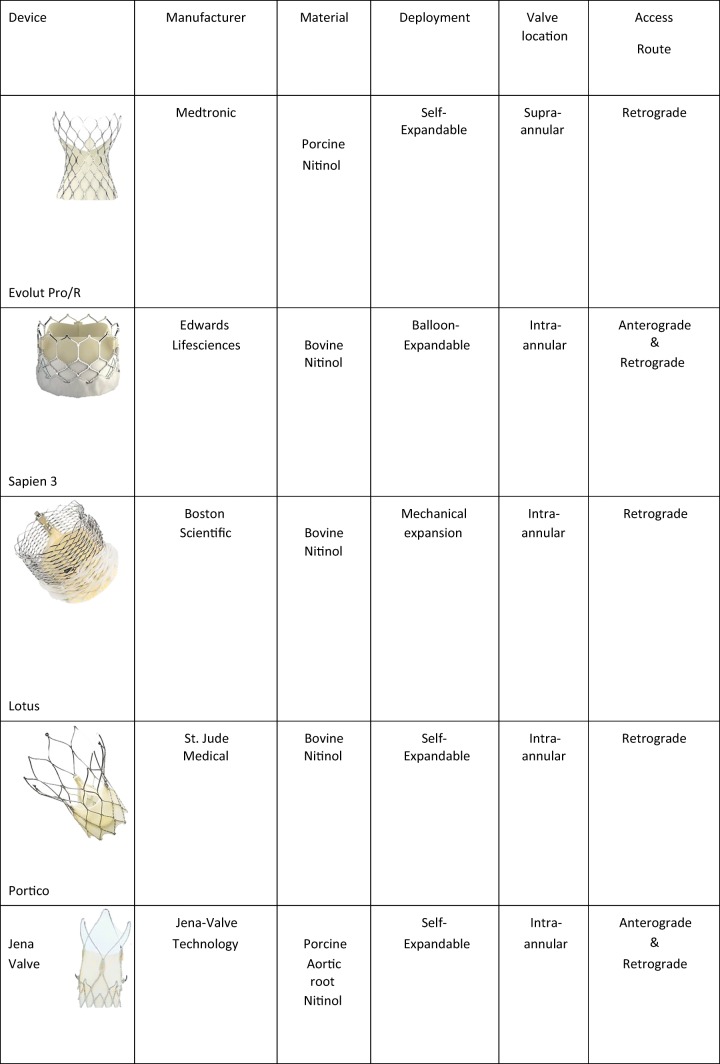
Overview of new generation TAVI devices

## Standardisation of scanning protocols

### Consensus statement

- *The CT acquisition protocol should at least include a contrast enhanced ECG-gated or triggered scan of the aortic root reconstructed with 1.0 mm or less slice thickness, preferably with several reconstructed phases but at least including a systolic phase.*
- *A contrast enhanced CT scan with a scan range that at least extends from the subclavian arteries to the superficial femoral arteries at the level of the femoral head is required.*
- *Both scans may be obtained from a single acquisition but in most cases two separate acquisitions (one for the aortic root and one for the vascular access) during the same session are preferable.*
- *Tailoring CT acquisition protocols to lower the required volume of contrast material prevails over radiation dose reduction given the fragile nature of the patient population and the need for high quality images, with the newer CT systems having the possibility to use one contrast bolus for evaluation of access route and valve area.*
- *MR can be used as an alternative to CT for TAVI planning but is more complex and may be considered in patients with severely depressed renal function given the availability of unenhanced MRI protocols.*

### CT

#### General scanner and acquisition requirements

CT scanning protocols should be optimised according to the available technology, considering that two separate subsequent acquisitions are often necessary to cover a large anatomic range from the subclavian to the femoral arteries, as well as an ECG-gated acquisition of the aortic root.

Images should be reconstructed at 1.0 mm or less to enable accurate multiplanar reformations. Therefore at least a 64-slice or Dual-Source scanner is required.

Although radiation dose reduction is always an issue, in the fragile patient population assessed for TAVI, reduction of iodinated contrast dose and avoiding the need for repeated contrast injection due to insufficient image quality is far more important and should prevail over radiation dose reduction.

Potential intrinsic renal frailty of these patients is, in fact, worsened by the need to use an additional contrast dose to perform fluoroscopy and angiography following CT examination, which may lead to higher risks of contrast-induced nephropathy (CIN) and acute renal failure [32].

An ESCR recommended scanning protocol has been summarised in a separate section of this article (Supplementary Material 1) for different commercially available single – and dual-source CT scanners with normal and high-pitch protocols.

#### CTA of the aortic root

A retrospectively ECG-gated or prospectively ECG-triggered CTA of at least the aortic root is mandatory for the motion-free evaluation of the left ventricular outflow tract (LVOT), annulus, sinus of Valsalva, sinotubular junction, ascending aorta and coronary ostia. The annulus undergoes conformational changes during the cardiac cycle; images are preferably obtained at least during systole [33]. For prospectively ECG-triggered scanning, the use of ECG-padding or a wide pulsing window is recommended so multiple phases can be reconstructed, increasing the likelihood of having at least one motion-free phase in case of arrhythmia. A narrow field of view centred on the aortic root should be used to increase spatial resolution. In practice, an acquisition protocol used for coronary imaging is usually a good starting point, requiring only further modification to include high-quality systolic images (e.g. adaptation of dose modulation).

Optionally, a non-contrast-enhanced acquisition of the aortic root is included, using acquisition parameters identical to a non-contrast CT for coronary calcium scoring (120 KV, 3-mm slices). This allows to calculate the calcium score of the aortic valve.

#### CTA of the aorta and iliac arteries

Scan range should at least extend from the subclavian arteries to the superficial femoral arteries at the level of the femoral head. Depending on the scanner hardware, this may be the same ECG-gated or ECG-triggered acquisition as for the aortic root. This approach, however, frequently results in a relatively high radiation and/or contrast dose compared with non-gated acquisitions. Since a non-gated acquisition is adequate to evaluate the aorto-femoral vessels, usually a second acquisition following that of the aortic root is used. For modern dual-source scanners, a single high-pitch acquisition triggered at imaging the heart during systole may be used but offers only one reconstruction phase of the annulus. This may be problematic in case of motion artefacts, with limited options to improve image quality afterwards. Despite not constituting a real late-phase acquisition, two subsequent acquisitions also help to differentiate circulatory stasis in a large left atrial appendage from a real thrombus.

#### Contrast administration/volume

Fast anatomic coverage and low KV (70–80 kV), imaging is especially recommended in this fragile patient group to allow for a reduction in the amount of contrast agent [34]. A single injection of contrast agent for both acquisitions is recommended. Around 50-ml contrast material in total at a flow rate of 3–4 ml/s is often sufficient but should be adapted to the capabilities of the CT system and the body habitus of the patient [34].

#### Medication

Nitroglycerine and beta-blockers are contraindicated in severe aortic stenosis and should not be administered prior to scanning.

### MRI protocol

MR has many potential advantages in TAVI planning [35, 36]. It quantitatively and radiation-free assesses aortic valve stenosis and regurgitation, coupled with accurate evaluation of the impact of valvular disease on ventricular function. MR can also provide all the measurements needed for the procedure, comparable to cardiac CT. Evaluation of aorta and iliaco-femoral arteries is also possible.

Additional strengths of MR imaging include the late enhancement evaluation of macroscopic fibrosis in aortic stenosis, the use of a gadolinium-based contrast medium which is significantly less nephrotoxic and produces less adverse reactions than its iodine-based CT counterpart and the ability to perform a non-contrast-enhanced study in patients with severely impaired renal function.

Nevertheless, its use is far less widespread for annular measurements compared with CT. Probable reasons include a technically more complex examination, a longer study time and a higher required degree of patient cooperation. Also, valve calcifications, while visible, are rendered with less detail, and no calcium quantification is possible should this be required. However, on a case-by-case basis, it may be considered over CT in patients with severely depressed renal function.

A simplified MR protocol should start with coronal and axial black blood ECG-gated half-Fourier fast spin echo images, acquired at end-expiration, for general chest evaluation.

Then steady state free precession (SSFP) cine images are acquired at end-expiration along 2-chamber, 3-chamber and 4-chamber long axis and short axis. Furthermore, two long axis cine images of the aortic root are obtained, the first in an oblique coronal plane and the second obtained from the first, along the plane passing through aortic root and ascending aorta. Finally, a stack of cine images are acquired orthogonally to the above two planes, covering the entire aortic root.

After these planes, mandatory for all the aortic and functional measurements, a different approach can be used, based on the patients’ renal function: if a contrast agent can be administered, a multi-step contrast-enhanced MR angiography (CE-MRA) is obtained, from aortic arch to proximal femoral arteries. This represents the fastest approach, as the scan time is less than 1 min, according to different sequences and acceleration factors.

In the presence of aortic valve stenosis or concurrent myocardial diseases, a late enhancement sequence of the left ventricle can be acquired, to assess macroscopic fibrosis.

In case of severe renal failure or known allergy to gadolinium chelates, thoracic and abdominal aorta and iliaco-femoral arteries can be assessed by means of different sequences: a 3D-SSFP navigator-echo and ECG-gated (so-called whole heart) sequence can be used for the thoracic aorta, while a non-contrast-enhanced MRA (flow-enhanced based or flow-independent based) can be used for aorto-iliac evaluation [37]. Alternatively, the use of an intravascular contrast (ferumoxytol) has been described, but is currently not widely available in Europe [38].

An ESCR MR recommended protocol for pre-TAVI evaluation is given in Supplementary Material 2.

## Required CT-derived measurements and imaging features before the procedure: recommended stepwise approach

### Consensus statement

- *The main elements of CT in annular sizing are:*

- *to define a cross-sectional double-oblique image orientation in the correct plane of the aortic annulus*
- *to obtain accurate and standardised measurements of different annular dimensions and height of coronary ostia*
- *to implement these measurements in the selection process of a TAVI candidate in order to have the optimal prosthesis-patient matching*

- *ECG-gated acquisitions are mandatory, with a preference for systolic measurements.*
- *Evaluation of all potential access routes for suitability is mandatory.*
- *For valve-in-valve procedures, simulated TAVI insertion is mandatory to assess potential coronary obstruction.*

Since direct measurements of the aortic root are not possible, imaging-based anatomic assessment forms an essential pre-procedural step, crucial not only to determine TAVI eligibility but also to select the optimal choice of device type and size and the best pathway for device delivery.

The general principle of pre-TAVI CT imaging is to provide motion-free high quality images of the aortic valvular complex and root (i.e. aortic valve annulus, commissures, sinuses of Valsalva [SOV], ostia of coronary arteries [OCA] and sinotubular junction [STJ]) combined with a large longitudinal coverage encompassing the entire aortic course between the proximal supra-aortic vessels and the ilio-femoral axes for access evaluation [39, 40].

High-quality ECG-gated CT images of the aortic root are mandatory, as measurements should be performed in the systolic phase as the annulus undergoes conformational changes during the cardiac cycle and is usually largest in systole [33]. However, image quality prevails over cardiac phase selection as, depending on patient- and technical-related factors, diastolic images may be of better quality providing more reliable measurements. ECG gating of non-cardiac anatomy is not routinely recommended, as it does not provide additional benefit. However, as CT technology evolves, newer vendor-specific CT protocols may be adapted by the local team in order to achieve the best image quality possible.

An overview of all required measurements is given in Table 4.

**Table 4:** Overview of required measurements

Anatomy	Component	Characteristics
Aortic valve		
	Cuspidity	Bicuspid/tricuspid/undefinable
	Valvular calcifications	Amount (absent to severe)/location/distribution
	Subvalvular calcifications	Present or not, location, amount
	Quantification of valve leaflet calcification	Use the Agatston method, only indicated in discrepant Doppler echocardiography results
Aortic annulus		
	short- and long-axis diameter (mm)	Systolic measurements preferred, ensure correct double-oblique annular plane orientation
	Perimeter (mm)	
	Area (mm^2^)	
Aortic sinus		
	Height (mm)	Requirements differ from type of THV and manufacturer
	Width (mm)	
	Distance from annular plane to coronary ostia (mm)	
	Diameter sinotubular junction (mm)	
Aorta		
	Maximum cross-sectional diameter of ascending aorta (mm)	
	Cross-sectional diameter at different levels (mm)	
	Wall characteristics	Amount and distribution of calcification, thrombus, ulcerative plaques, other findings
Access route		
	Diameter subclavian and common carotid arteries (mm)	Minimal luminal diameters are required
	diameter of brachiocephalic trunk (mm)	
	Diameter of common and external iliac arteries (mm)	
	Diameter of common femoral arteries	
	Wall characteristics	Amount and distribution of calcification, thrombus, ulcerative plaques, other findings
	Left ventricular apex	Myocardium characteristics, presence of thrombus, other findings
	Ascending aorta	Wall characteristics, especially anterior and antero-lateral wall for transaortic access

### Essential aortic root assessment

**Fig. 2:**
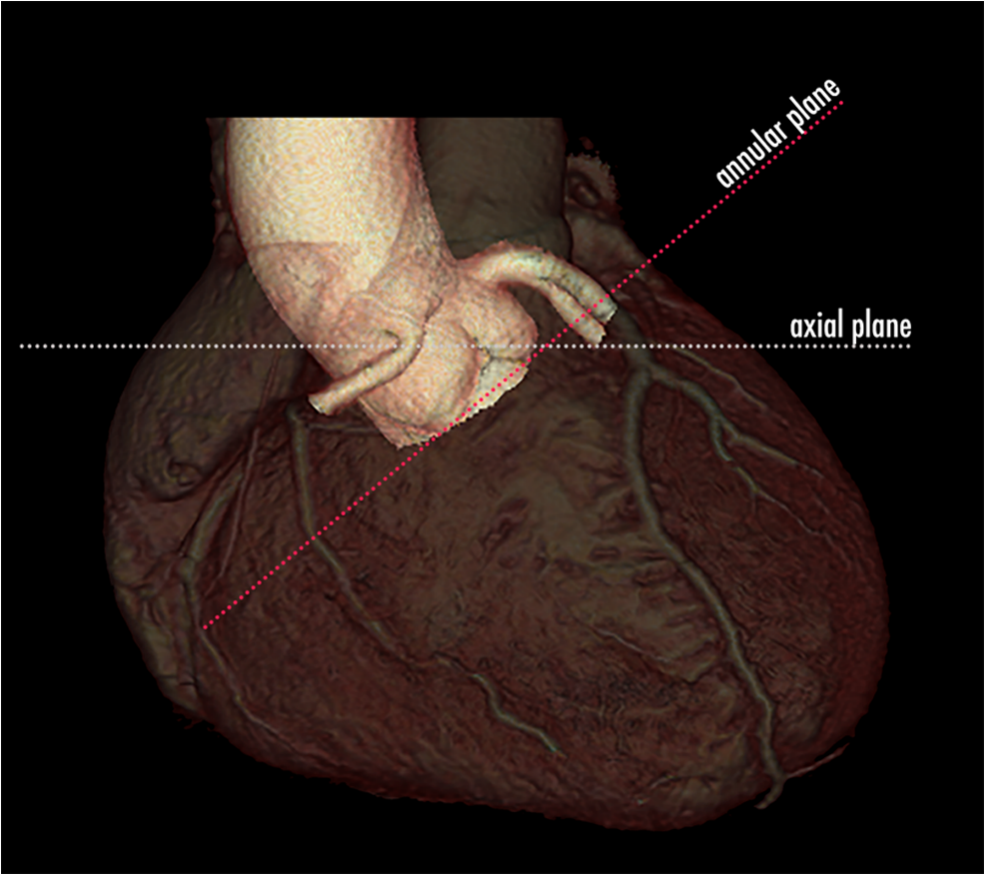
3D volume rendering CT image of the heart containing the aortic root. The aortic root has a double-oblique orientation within the heart. Therefore, standard orthogonal imaging planes, like the axial plane indicated with the dotted white line, are not suitable to correctly visualise the aortic root and containing structures. For this reason, intrinsic 3D imaging modalities like CT are necessary to correctly assess the aortic root and annulus and obtain accurate measurements

The aortic root extends from the left ventricular outflow tract (LVOT) to the sinotubular junction and has a rather central and double-oblique orientation in the heart (Fig. 2). As such, it is ideally visualised using a 3D imaging modality. Standard coronal, sagittal or even single-oblique image reformations are therefore not considered suitable and lead to incorrect measurements.

Assessment of the aortic root should include a description of the aortic valve morphology, and measurement of different annular dimensions at different cross-sectional levels of the aortic sinus.

#### Aortic valve cuspidity

**Fig. 3:**
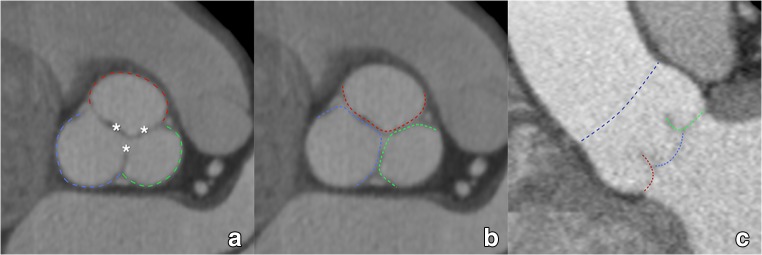
The aortic sinus contains the aortic valve, in most patients composed of three leaflets (asterisk in **a**, coloured dotted lines in **b**, **c**). They are named according to the adjacent sinus of Valsalva (**a**): right coronary sinus (red), left coronary sinus (green) and non-coronary sinus (blue). As such, we distinguish (**b**, **c**) a right coronary cusp (red), left coronary cusp (green) and non-coronary cusp (blue). The dotted dark blue line in (**c**) indicates the sinotubular junction, marking the roof of the aortic sinus

**Fig. 4:**
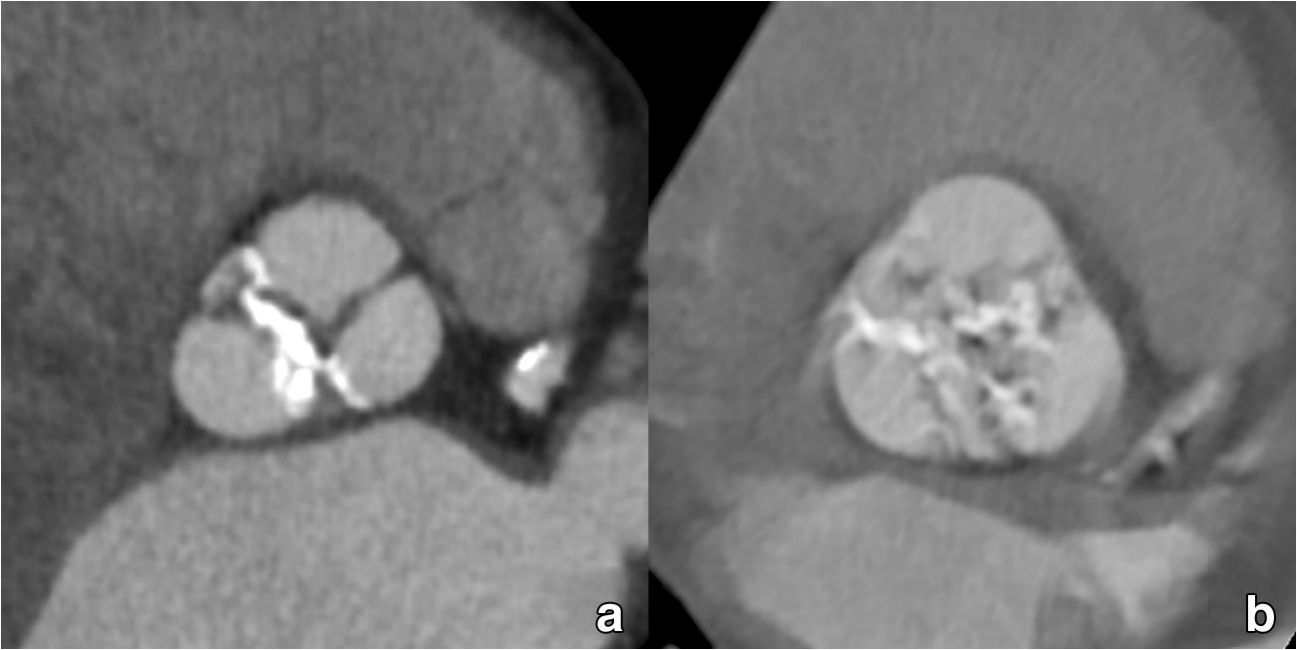
Most TAVI candidates will present with an aortic valve containing significantly calcified valve leaflets. The majority of patients will have a clearly identifiable tricuspid aortic valve (**a**). However, a significant portion will have a bicuspid aortic valve, which is an important feature to report as its presence is associated with some specific complications. However, in some cases, valve cuspidity can be difficult to assess in heavily degenerated valves, where extensive calcification can make differentiation between tricuspid and (functionally) bicuspid valves difficult (**b**)

The aortic sinus contains the aortic valve, in most patients composed of three distinctive aortic valve leaflets (Fig. 3). In TAVI candidates, this valve is often significantly calcified. The number of discernible valve leaflets or valve cuspidity should always be commented on, although it may sometimes be difficult to assess in heavily calcified valves (Fig. 4). However, detection of a bicuspid valve is relevant because AS is a known complication of a bicuspid aortic valve (BAV), where valve remodelling and degeneration occur faster and more often than in tricuspid valves. As such, BAVs account for about half of all aortic valve replacements for AS and are by extension therefore not rare in TAVI candidates [41]. While a bicuspid valve morphology is not a contraindication for a TAVI procedure, it might increase procedural complexity and is associated with a higher permanent pacemaker rate after implantation [42, 43]. Furthermore, BAV presence in referred pre-TAVI candidates may also increase in the future, as new studies and guidelines provide further support for potentially expanding TAVI indications to patients with intermediate surgical risk and of potentially younger age, in which BAV is thought to be more common [7, 44].

#### Amount, location and distribution of valvular calcifications

**Fig. 5:**
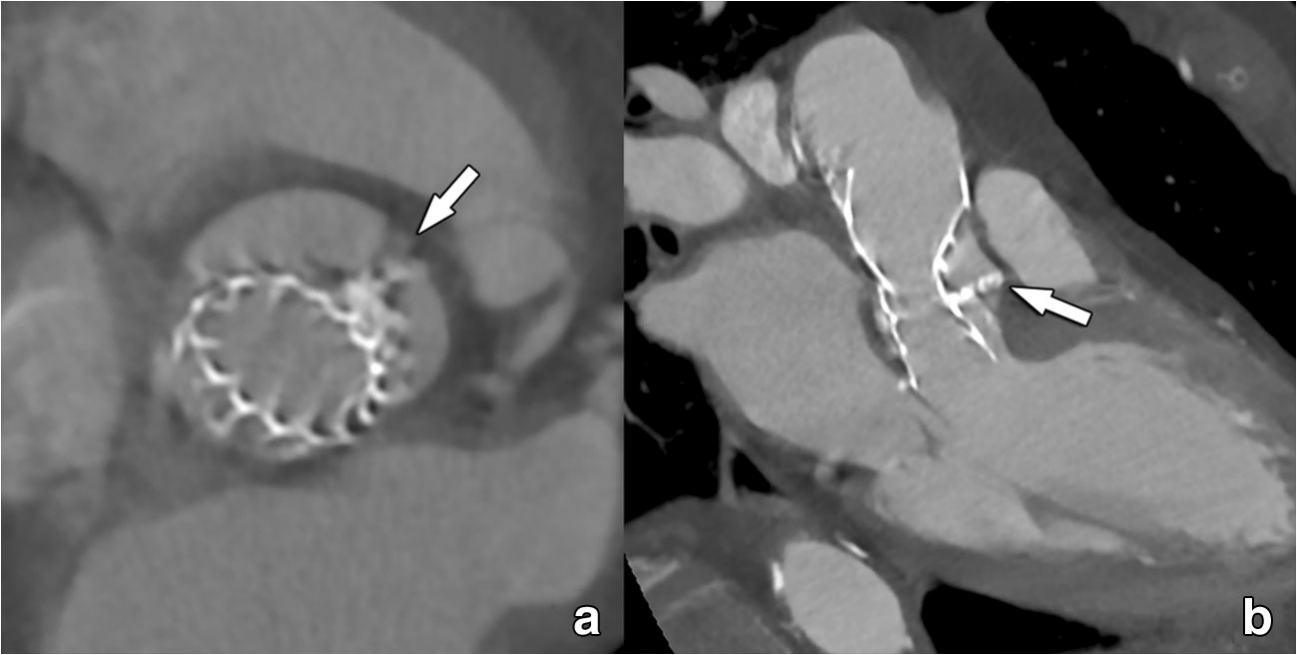
Incomplete and asymmetric deployment of a self-expandable THV due to interposition of extensive native leaflet calcifications (arrow in **a**, **b**) between the prosthetic valve and the wall of the aortic sinus. Severe calcifications can complicate prosthesis deployment as in this case, leading to a deformed THV. Nevertheless, caution should be taken when extrapolating morphological findings into a potential dysfunction. While in this case the residual gap between the THV and the aortic wall would suggest a severe paravalvular leakage, this was not the case on Doppler echocardiography examination, with the calcification apparently acting as an additional seal. Valvular function was acceptable, and no further intervention was deemed necessary

**Fig. 6:**
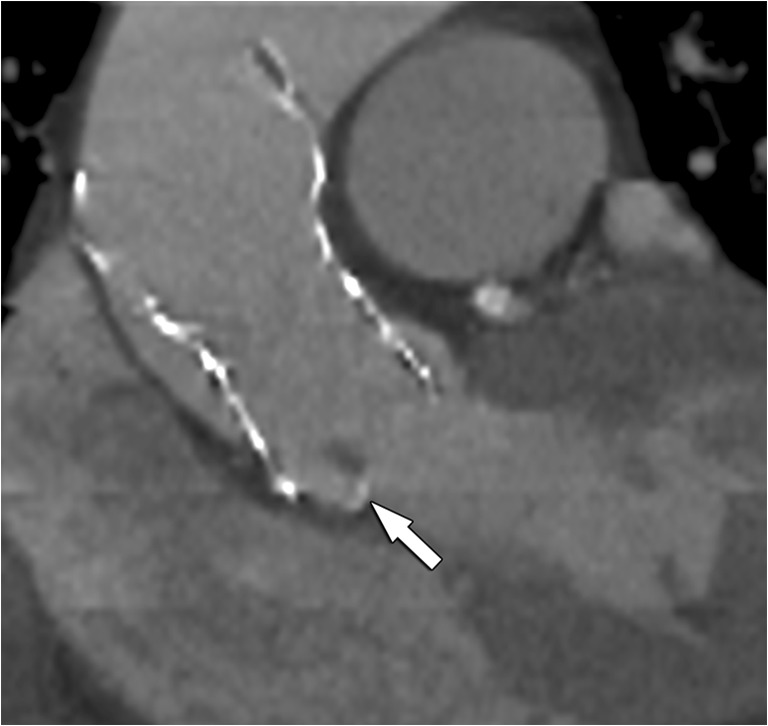
Incorrect positioned THV, which is tilted and does not fully extend into the aortic annulus. As such, parts of the native right aortic valve leaflet is protruding into the inflow part of this self-expandable THV (arrow), causing a residual valve gradient on Doppler echocardiography. CT is very useful in detecting the cause of THV dysfunction in cases where Doppler echocardiography does not provide an answer. In this case, function was improved after balloon dilatation of the inflow part of the THV, further crushing the remaining valve leaflets against the adjacent aortic wall

**Fig. 7:**
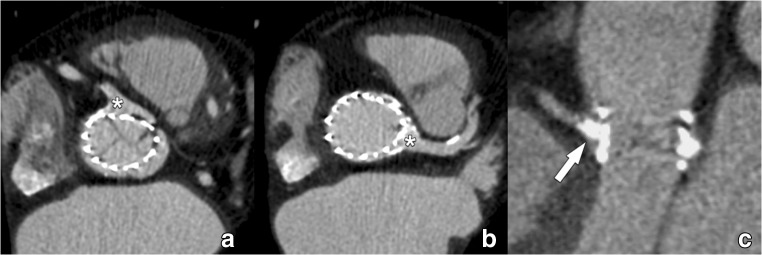
Relation between the coronary ostia and the deployed THV. Both self-expandable and balloon-expandable THVs are designed not to obstruct the coronary ostia, with self-expandable Corevalve and Evolut protheses extending into the ascending aorta by design, leaving the coronary ostia open (**a**, **b**). When coronary obstruction occurs, it is not by the THV but secondary to displaced calcified native leaflet remnants that migrate during deployment of the THV in the aortic sinus to the vicinity of the coronary ostia. Nevertheless, while CT can detect these migrated calcifications in or near the coronary ostia (**c**), the evaluation of luminal patency is less obvious, mostly dependent on local expertise and the quality of the CT scanner used

Due to mechanic effects, excessive or eccentric “landing zone” calcifications may hamper appropriate prosthesis anchorage, leading to gaps between the prosthetic valve and aortic annulus (Fig. 5). This may lead to the possible occurrence of paravalvular leak, which however cannot be further assessed using CT [45]. Similarly, severe aortic valve calcification is a known risk for annular rupture with balloon expansion, prosthesis dislodgement (Fig. 6), coronary ostia obstruction (Fig. 7), calcific embolism and stroke [45, 46].

**Fig. 8:**
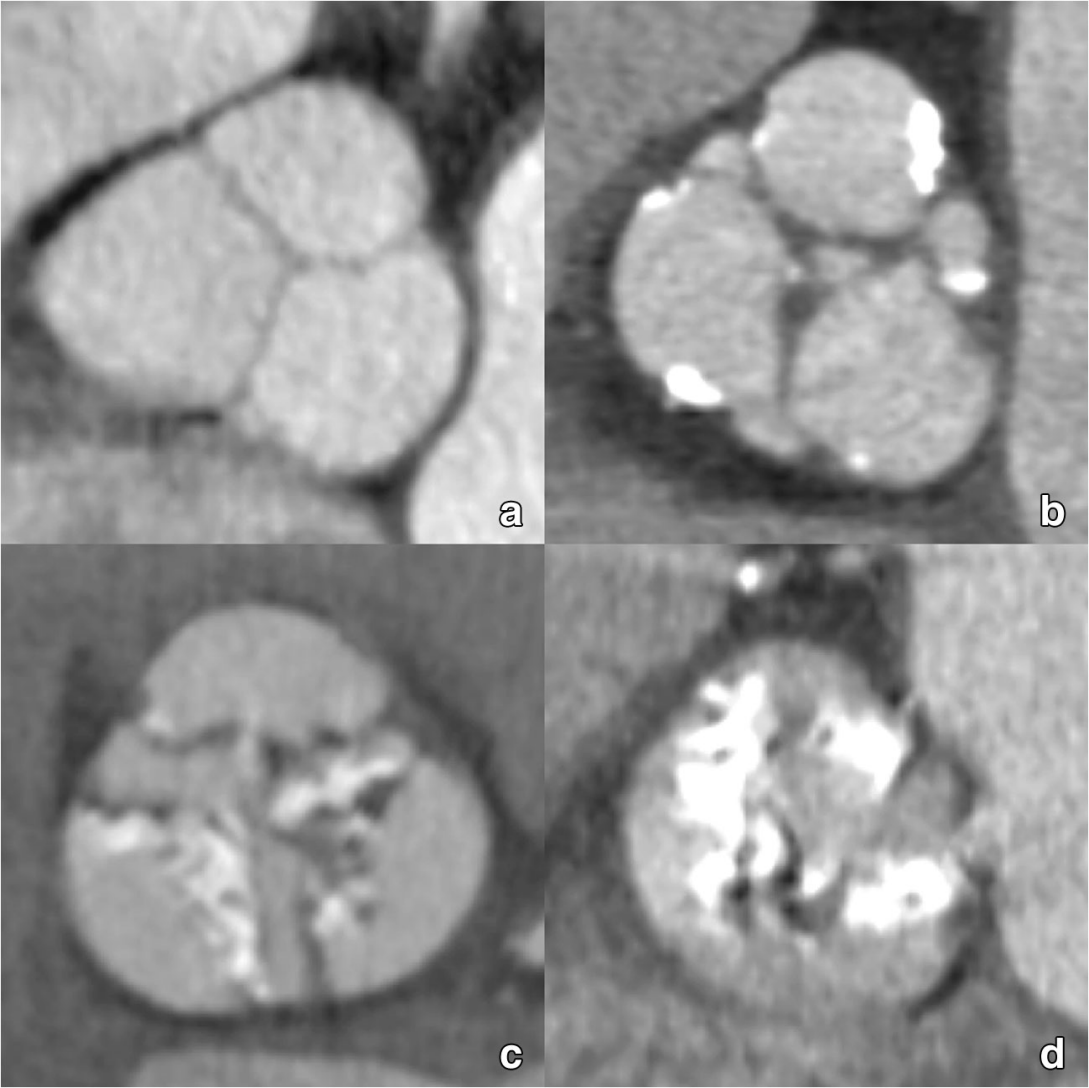
CT images of the aortic valve illustrating different degrees of valvular calcification depositions: none (**a**), mild (**b**), moderate (**c**) and severe (**d**). Also note the differences in the affected cusps and distribution (leaflet edges, commissures and attachment sites)

**Fig. 9:**
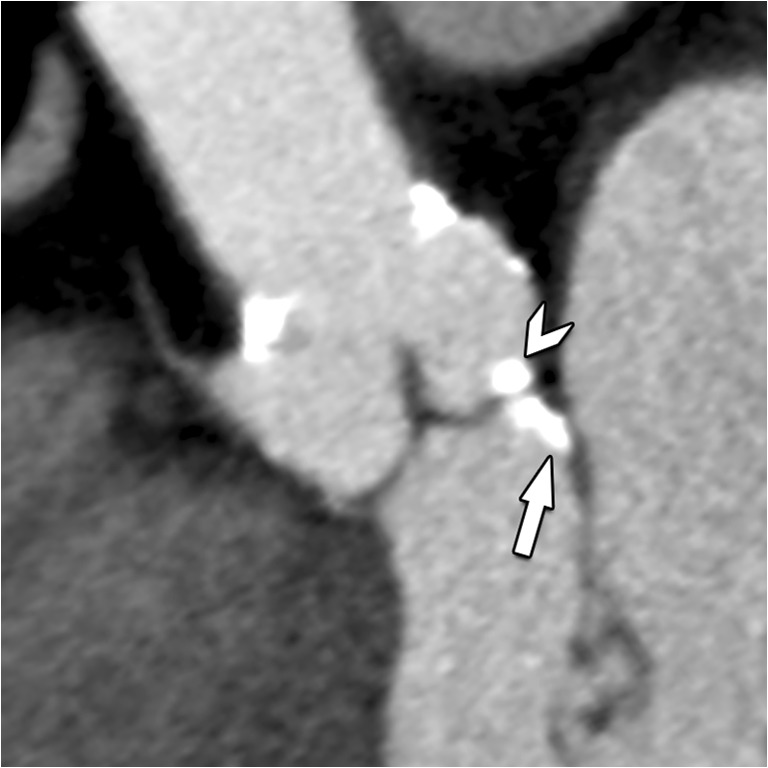
Double-oblique CT image of the aortic root in a TAVI candidate. While most calcification will be on a supra-annular level (arrowhead), occasionally, calcifications can also be found on an infra-annular subvalvular level (arrow). Reporting of these latter calcifications is important, as they can hamper proper deployment and attachment of the prothesis

Besides quantitative assessments for diagnosis of AS, which can be performed with CT using calcium-scoring techniques in selected cases, we recommended to categorise calcifications as symmetric or asymmetric and to visually score the amount of valvular calcium depositions (mild, moderate or severe), hereby considering the number and position of affected cusps (leaflet edges, commissures, and attachment sites) (Fig. 8) and the distribution pattern (diffuse vs. focal, subvalvular) (Fig. 9). A proposed classification for pre-TAVI valvular calcifications visual grading is reported in Table 5.

**Table 5:** Visual description and grading of aortic valve calcifications

Semi-quantitative pre-TAVI grading of valvular calcifications	
Absent	No calcifications
Mild	Small isolated focal spots not involving commissures and attachments sites
Moderate	Large confluent calcifications affecting 2 cusps or Small isolated focal spots at the level of all commissures and attachments sites
Severe	Large confluent calcifications affecting all cusps

Large tables, see separate document

#### Planimetry of the annular plane (see Supplementary Material 2–4)

**Fig. 10:**
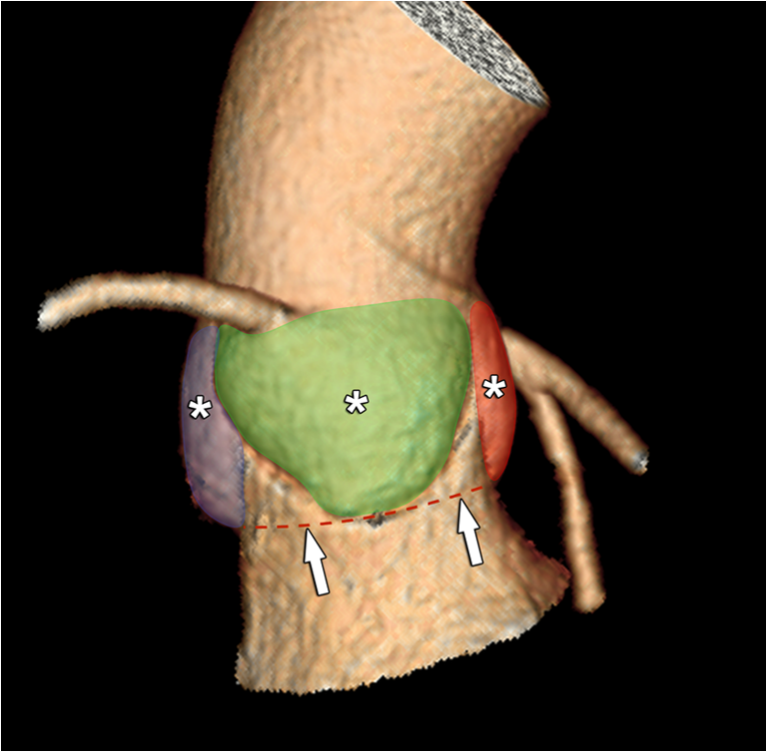
3D CT image of the aortic root containing the sinuses of Valsalva (asterisk). As the aortic valve leaflets extend within these sinuses up to the sinotubular junction, connecting their most basal attachment sites forms a virtual ring which is named the aortic annulus (red dotted line, arrows). It also marks the transition to the LVOT

In contrast to previous beliefs, the aortic annulus is not a real anatomic structure. It is better thought of as a descriptive term commonly used by surgeons to indicate the *virtual* aortic wall ring formed by connecting the nadirs of the attachment sites of the aortic valve leaflets (at the basal portion of the sinus of Valsalva) (Fig. 10). As such, it contains no fibrotic tissue. The *annular plane* of the aortic annulus is therefore defined by connecting these three lowest insertion points of the aortic valve leaflets.

**Fig. 11:**
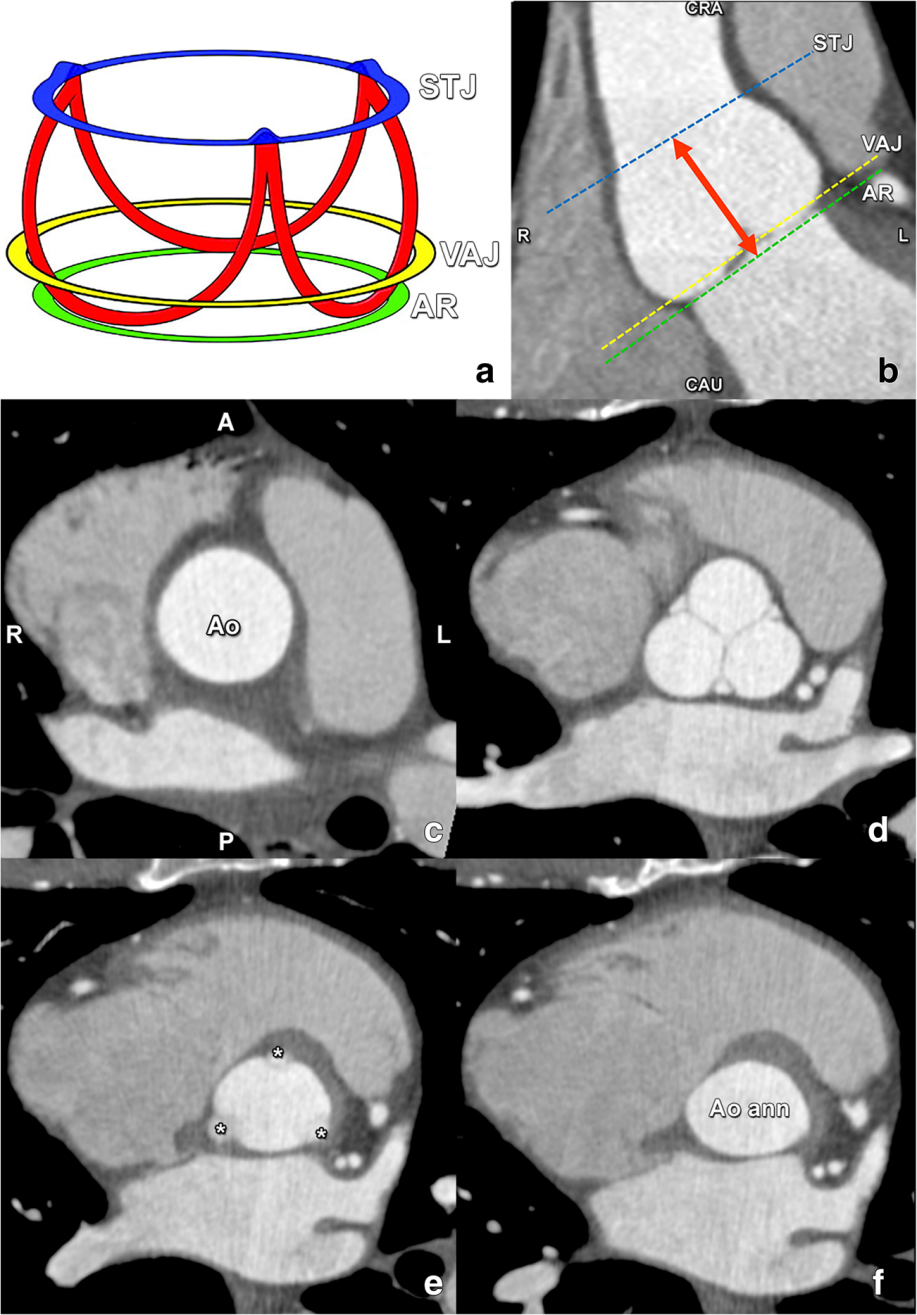
**a** Schematic drawing illustrating the crownlike suspension of the aortic valve leaflets within the aortic root extending across the length of the aortic sinus (**a**). AR, virtual annular ring representing the annulus (green), formed by joining the basal attachments of the aortic valve leaflets; STJ, sinotubular junction (blue); VAJ, ventriculo-arterial junction (yellow). Red, aortic leaflet insertion sites in the sinus of Valsalva forming a crownlike ring. **b** Coronal contrast-enhanced CT image demonstrates the levels of the sinotubular junction (STJ) (blue line), ventriculo-arterial junction (VAJ) (yellow line) and annular ring (AR) (green line). Double-headed arrow, anatomic range of the sinuses of Valsalva. CAU, caudal; CRA, cranial. **c**–**f** Double-oblique reformatted images further clarify the changing shape of the aortic root contour. **c** The sinotubular junction forms the top of the crown, where the outlet of the aortic root in the ascending aorta (Ao) is a true circle. A, anterior; P, posterior; L, left; R, right. **d** The aortic root gradually becomes less circular, with a more cloverleaf shape at its midportion (i.e. at the sinuses of Valsalva). At this level, the aortic valve leaflets are clearly seen. **e** The aortic valve leaflets (asterisk) are just barely visible at the level of the ventriculo-arterial junction, where the left ventricular structures give rise to the fibroelastic walls of the aortic valvar sinuses. Note that the aortic root contour is now becoming increasingly ellipsoid. **f** The bottom of the aortic root is formed by the virtual ring, or aortic annulus (Aoann), which has an oval shape in most patients. Reused from reference [39], with permission

The aortic valve has a complex semilunar, crown-shaped three-dimensional morphology, extending from the sinotubular junction to the basal attachment plane of the aortic valve leaflets (the so-called annular plane), located just below the ventriculo-arterial junction (Fig. 11). Note that the lowest insertion of the right coronary cusp leaflet is often inferior to the left and non-coronary cusp leaflets.

The virtual ring addressed as aortic annulus was traditionally assumed to be always circular. However, while on cross-sectional imaging the aortic root contour is indeed practically circular at the level of the STJ, it assumes a more clover-leaf shape at the level of the aortic sinus, often becoming oval to ellipsoid at the annular plane and the LVOT (Fig. 11). In heavily calcified anatomy with severe aortic stenosis/insufficiency, the cross-sectional shape can be even more complex, difficult to define and not comparable to geometric assumptions.

Exact measurements are crucial for a successful TAVI procedure, as small differences in the choice of a measurement plane in the aortic root and choice of start- and endpoint of the selected diameter can produce notably different results, influencing the choice of THV size. Therefore, high-quality images are essential in order to provide reliable measurements.

The main elements of CT in annular sizing are:

- Obtaining a cross-sectional image orientation in the correct plane of the aortic annulus
- Correctly and standardised measuring the annulus using different methods
- Implementing these measurements in the selection process of a patient-specific THV size.

**Fig. 12:**
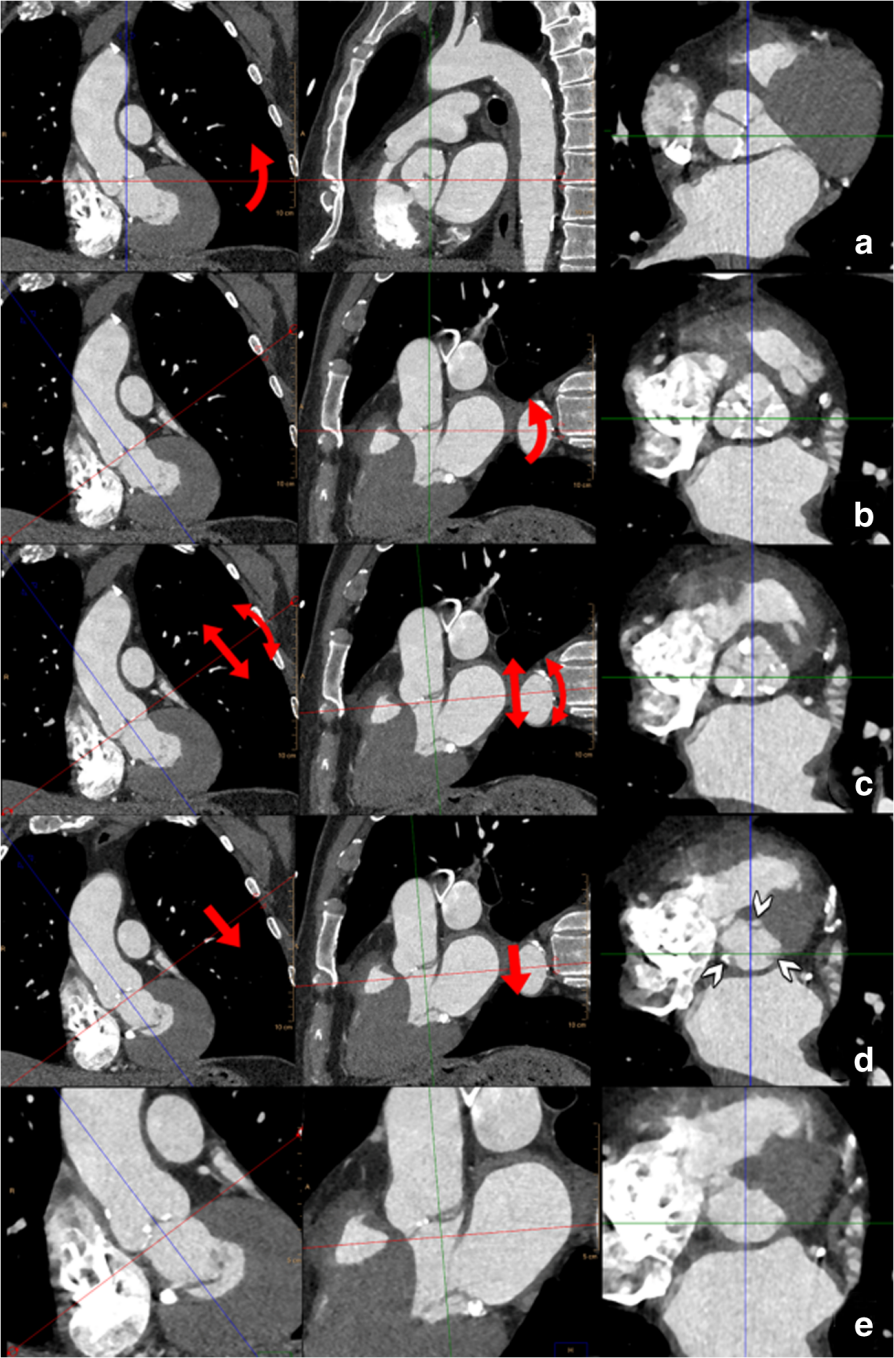
For all measurements of the aortic root as illustrated in Figs. 12, 13 and 14, the use of a (simple) multiplanar reconstruction viewer is mandatory. The three imaging planes should be perpendicular to each other at 90° angles and the reference lines should be “locked” so rotating one reference line automatically rotates the other planes. Care should be taken to have the screen layout setting in such a way that all three imaging planes (starting with axial, coronal and sagittal) are visible simultaneously. The CTA dataset (preferably a systolic phase) is loaded into the viewer. **a** First, in the coronal plane the aortic valve is located and the centre of the reference lines is placed approximately at the centre of the aortic valve. In the coronal image plane, the references lines are rotated so one of the two lines is at approximately 45° to the horizontal level. This results in the images seen in **b**. In the plane that was the original sagittal reconstruction (middle panel in **b**), the reference line is also rotated to be approximately parallel to the aortic valve. This generally provides a pretty good imaging plane that is perpendicular to the aortic valve (right panel in **b**). The essential step (illustrated in **c**) is to scroll up and down through this image stack (as indicated by the straight arrows in the other views in **c**) and determine if all three aortic valve cusps are seen symmetrically in each image (i.e. scrolling from the level of the LVOT to the aortic valve, the three cusps should appear symmetrically and simultaneously in one image). This is often not yet the case. By tweaking the angulation of the plane by slight rotation of the crosshairs in the other views (as indicated by the curved arrows in **c**) while assessing its effect on the symmetry of the valve leaflets in the in-plane image is needed to have the cusps appear symmetrically. Once this has been established, by scrolling through the image stack in-plane with the aortic valve towards the LVOT, the leaflets will increasingly appear smaller and closer to the aortic wall (**d**, see arrowheads in right panel). The first image just below the level of the lowest image (i.e. closest to the LVOT) that no longer shows the leaflets is selected and represents the annulus (**e**)

A recommended stepwise approach to get a proper anatomic orientation of the tricuspid aortic annulus is displayed in Fig. 12 (see Supplementary Material 2–3). Currently, there is no consensus on how to define the annular plane in bicuspid aortic valves as the basal attachments of the two leaflets provide only two landmarks out of a necessary three to define a plane in space.

**Fig. 13:**
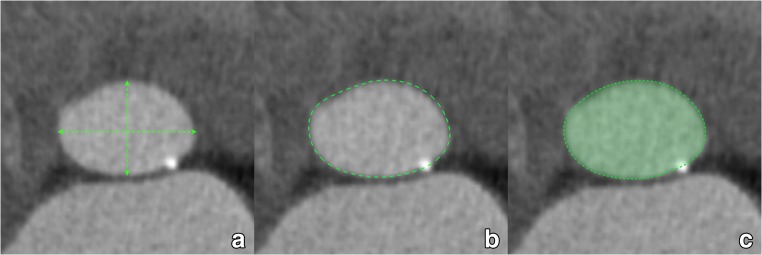
The annular plane image obtained through the steps outlined in Fig. 12 is used for the measurements. The long- and short-axis diameters are measured using a simple distance tool (**a**). In the annular plane, the circumference of the annulus is traced using a planimetry tool (**b**). Most software systems then automatically display the area, perimeter and area-derived diameter of the traced area (**c**). Alternatively, parameters can be calculated as described in the text

Once a suitable plane has been obtained, several annular measurements can be taken. We propose the calculation of the mean annular diameter using the three following methods, as stated preferentially based on systolic images as they provide the largest possible annular dimensions (Fig. 13).

First, we obtain annular cross-sectional long- (DL) and short-axis (DS) diameters.

Then, the annular perimeter is manually tracked using a planimetry tool on a workstation, after which area A and circumference C of the aortic annulus are derived by the workstation software. Finally, the mean annular diameter D is calculated based on these different measurements. For the cross-sectional-derived mean diameter DCS, this is done by simple averaging (DCS = (DL + DS)/2). The area (DA) and circumference-derived (DC) effective diameter are calculated as follows: DA = 2 × √(A / π ), and DC = C/ π.

It is however important to realise that DC and DA are calculated under the assumption of full circularity of the annulus after device deployment, a feature almost exclusively found in deployed balloon-expandable valves. The discrepancy between these three measurements (DCS, DA and DC) will therefore increase with remaining annular eccentricity, most notably in the circumference-based method. This further underlines the important concept that transcatheter valve size selection is closely tied to the type of device used and that sizing algorithms are not strictly interchangeable.

When borderline results not allow to choose between two different potential prosthesis sizes (e.g. 23 vs. 26 mm), it is recommended to do a blinded complete re-measurement in order to acquire more certainty, together with all available imaging data from different sources.

### Additional recommended measurements in the aortic root

#### Minimum distance of the annulus to the left and right ostium of the coronary arteries (see Supplementary Material 5–6)

Coronary obstruction secondary to migrated calcified and non-calcified native valve components during device deployment is a rare procedural complication with a reported incidence of 0.8%, increasing to 3.5% in valve-in-valve procedures [47, 48]. It usually occurs during the procedure but has been reported up to 24 h after device deployment [49, 50]. Patients with a more susceptible anatomy for this complication have a combination of a low-lying ostia of the coronary arteries (OCA) with a large native aortic valve leaflet.

**Fig. 14:**
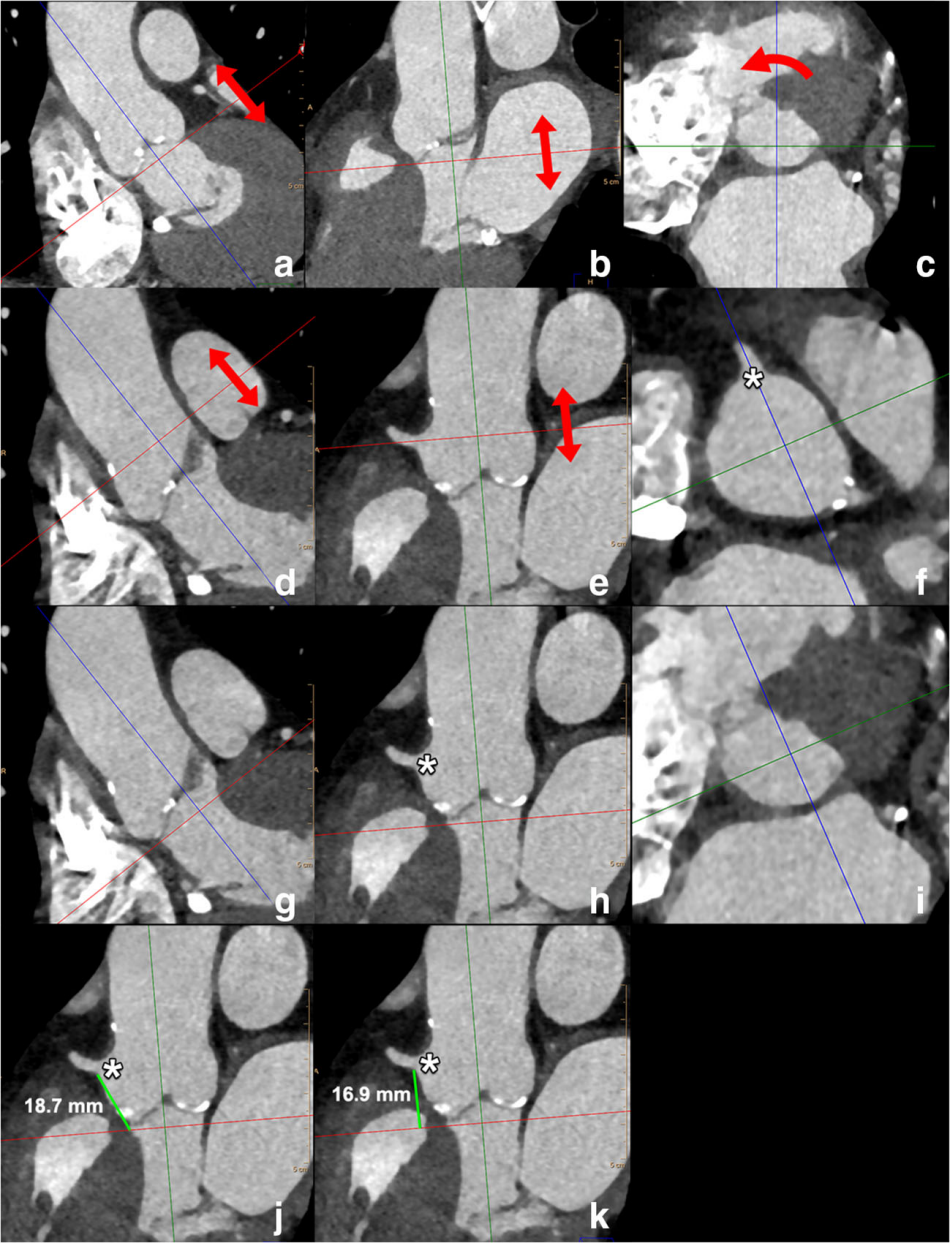
Standardised way to perform measurements of the distance of the annular plane to the ostium of the right coronary artery and left main. The starting points are the annular plane images (obtained through the steps outlined in Fig. 12) as displayed in **a**–**c** above. In this image stack, in plane with the annulus, the origin of the RCA is located by scrolling through the images in the direction of the aorta (arrows in **a**, **b**). Subsequently, the reference lines are rotated (curved arrow in **c**) in such a way that one of the reference lines passes through the RCA ostium (asterisk in **f**); then, by scrolling toward the LVOT (arrows in **d**, **e**), the annulus plane is again displayed (**i**) and in one of the two other panels (**h**), the origin of the RCA is visible (asterisk in **h**), as well as the reference line which corresponds to the annulus plane level (red line in **h**). The distance is measured as the distance of the lower border of the RCA ostium (asterisk in **j**, **k**) to the attachment of the coronary cusp (**j**) or perpendicular to the reference line of the annulus plane (**k**). For the distance of the annulus to the left main, the steps mentioned above are repeated

Distance should be measured by tracing a perpendicular line connecting the inferior edge of OCA with the aortic annulus plane, as the risk of obstruction is considered to be low if this height is more than 10–14 mm [39, 51]. Minimum distance to OCA should also obviously be calculated in relation to the length of the aortic valve cusps. A recommended stepwise approach to measure the minimum distance of the OCA is displayed in Fig. 14.

#### Largest dimensions of the aortic sinus and sinotubular junction (see Supplementary Material 7–8)

The largest diameter of the aortic sinus diameter and height should be assessed on a double-oblique projection. The sinus of Valsalva acts as a reservoir for the displaced native aortic valve calcifications after device deployment. The necessary dimensions of the aortic sinus are specified by the manufacturer of the specific device that will be implanted and varies from model to model.

#### Determination of optimal c-arm angulation

There is a remarkable individual variation of anatomic position of the aortic valve, which is usually projecting with a slight degree of caudal angulation in the right anterior oblique (RAO) projection and cranial angulation when in the left anterior oblique (LAO) [52]. During the procedure, the correct tube projection has to be determined in order to define the optimal fluoroscopic orientation consistent with an orthogonal view of the aortic valve plane. More specifically, a view with the origin of the right coronary artery pointing towards the viewer and the inferior margins of the three coronary cusps projecting at equal size and distance to each other with the right coronary cusp in the middle, the left coronary cusp to the left and the non-coronary cusp to the right. Without 3D imaging guidance, this would require multiple aortograms, increasing not only procedural time but also contrast volume, potential contrast-induced nephrotoxicity and radiation dose.

However, imaging-based prediction of the correct aortic annulus angle projection can reliably be derived from pre-procedural CT data, increasing procedural efficacy [53]. Therefore, we recommend providing this information in every report.

Nevertheless, a major limitation of CT-based prediction of aortic annulus projection is the assumption that a patient’s position would be comparable between the CT acquisition and the actual procedure, which is not always the case.

### Measurements for valve-in-valve procedures

For the valve-in-valve procedures, the size of the in situ surgical aortic valve prosthesis determines the maximum TAVI size that can be implanted. Often this is known from the surgical report. If they are not known, the type and size can be deducted from the CT appearance and standardised measurements [54, 55]. The main concern with valve-in-valve procedures is obstruction of the coronary artery ostium by the leaflets or struts of the surgical aortic valve and is much more common than in regular TAVI.

To simulate the effect of TAVI implantation on the coronary arteries, a circular region of interest/cylinder with the same diameter of the TAVI valve to be implanted is drawn at the level of the coronary artery ostium [54]. In this simulated TAVI implantation, the distance of the coronary ostium to the virtually implanted device can be measured. The thresholds for a safe minimal distance have not yet been defined or validated in large cohorts but several millimetres are considered to be necessary at least.

### Evaluation of the access route

Before actual THV deployment, the prosthetic heart valve has to be transported to the aortic root using a non-surgical approach. Several options are currently available.

**Fig. 15:**
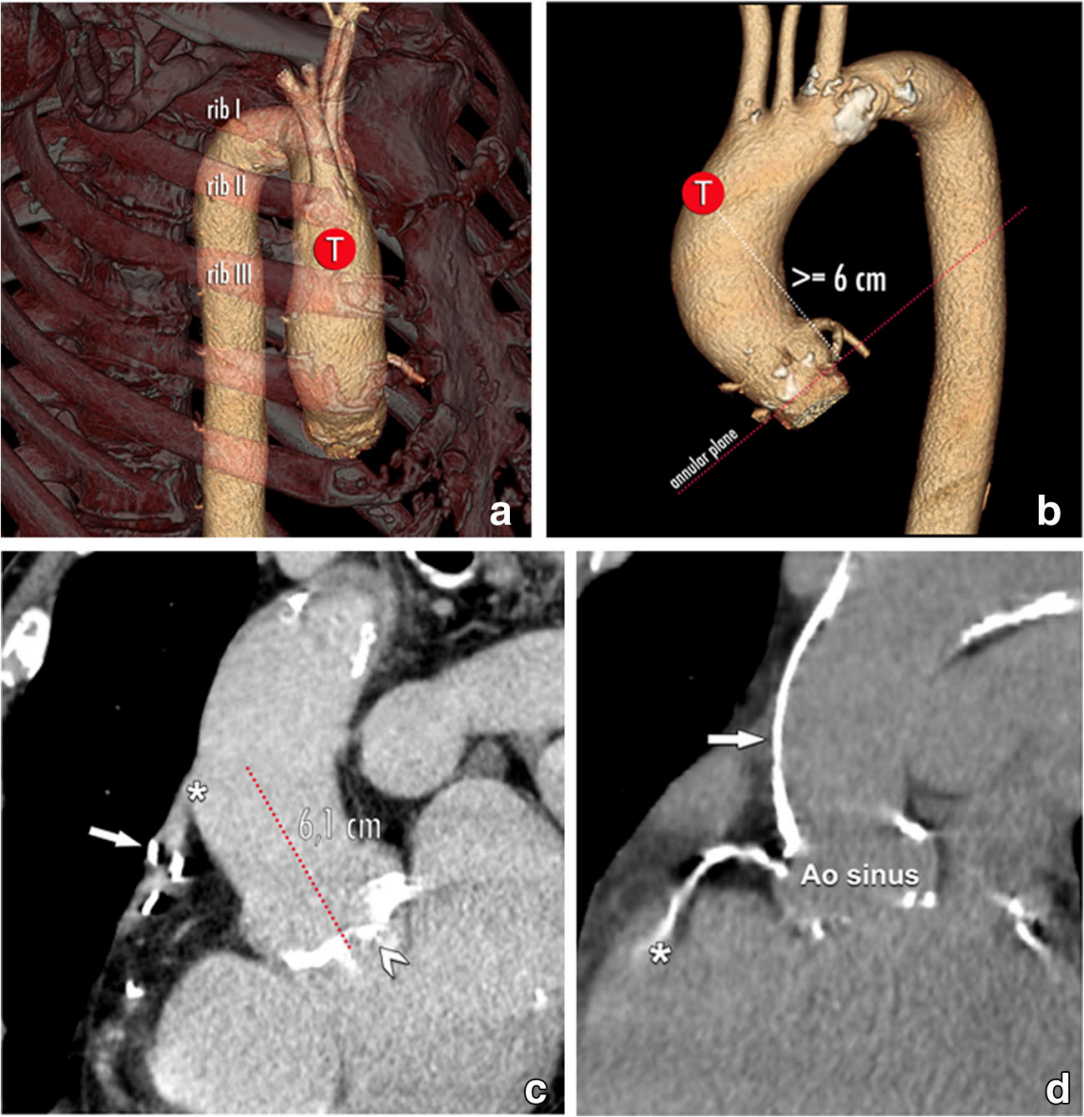
The target point (T) in case of a transaortic THV delivery. When the usual endovascular or transapical delivery routes are not possible, the transaortic pathway offers an alternative for patients in which no other access is possible. The recommended entry point for self-expandable THV is located at least 6 cm above the level of the aortic annulus (annular plane in red dotted line). Furthermore, the status of the aortic wall around this location needs to be scrutinised, as e.g. extensive calcification increases procedural feasibility and risk, and may as such make this access path unsuitable. However, even for transaortic access caution is needed. The TAVI candidate might have previous coronary bypass grafts, including a venous bypass over the RCA (arrow in **c**). The origin of this bypass (asterisk in **c**) might be near the targeted entry point, in this case about 6 cm above an annular plane with a heavily calcified aortic valve (arrowhead in **c**). Also, the anterior wall of the ascending aorta may show extensive calcification (arrow in **d**), making transaortic access impossible. Note incidental visualisation of an extensive calcified RCA (asterisk in **d**)

Arterial transfemoral access remains the approach of preference for all devices. Alternative entry points through the subclavian, common carotid and brachiocephalic artery are also possible for both BE and SE THV. SAPIEN devices additionally allow a left ventricular transapical approach. Finally, many centres have increasing experience using a minimally invasive transaortic pathway using a mini-sternotomy, applicable to both types of valves. The entrance point for transaortic access is about 6 cm above the annular plane (Fig. 15). All except the transfemoral approach require a surgical incision for initial access.

Regarding access sites, THVs come with a custom-made and device-specific delivery system for the transportation of the prosthetic valve. Different sheath sizes exist depending on the manufacturer and the production version of the device. Ideally, the minimal native vessel size should be larger than the outer diameter of the chosen delivery sheath. As such, smaller profile sheaths and delivery catheters improve procedural safety and expand patient eligibility. Currently, both Medtronic and Edward Lifesciences have 14F sheaths (16F for 29 mm Sapien 3 valve and 34 mm Evolut R valve) for transfemoral delivery. Depending on the chosen delivery system and THV size, the minimal vessel diameter can be as low as 5.5 mm. Delivery catheters are nevertheless the subject of intense research and have been continuously improved ever since their introduction, with other systems currently being developed by different vendors.

**Fig. 16:**
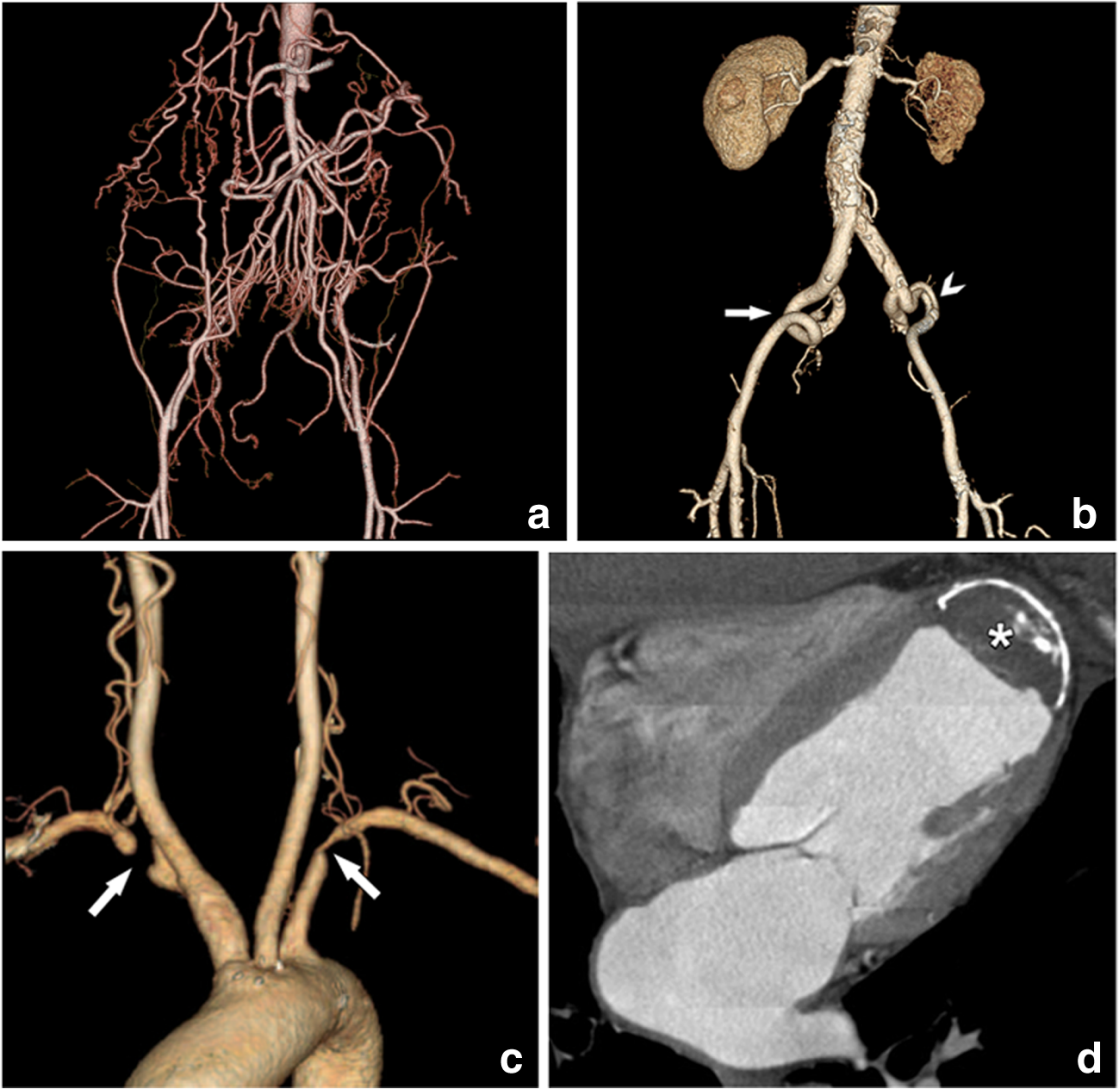
Compromised delivery paths. A safe endovascular trajectory is needed for safe transportation of the THV to the aortic root. CT is in his respect an essential tool in order to avoid vascular complications and guide to the intervention through the safest possible passage. Potential complications may arise due to luminal narrowing or even chronic iliac artery occlusion with extensive collaterals (**a**) and pronounced vascular tortuosity and kinking (arrows in **b**). In this last case, there is an additional short dissection in the left external iliac artery (arrowhead) due to a previously performed conventional coronary angiography. For these patients, the preferred transfemoral access approach is therefore not possible, and other options have to be considered. Nevertheless, other access paths may also pose significant challenges, like bilateral subclavian artery narrowing and occlusion (arrows in **c**), and the presence of post-infarct thrombus and wall calcification in the left ventricular apex (asterisk in **d**), making a transapical approach with a balloon-expandable valve impossible. Therefore, vascular access examination must include all anatomic possible entry points for a full assessment of the different options

As can be expected, a larger sheath size (22–24F) has been associated with a higher incidence of vascular complications varying from 23 to 31% compared with smaller systems (1.9–13.3%). Known risk factors which should be looked for include (Fig. 16):

- Amount and distribution of atherosclerotic (specifically circumferential) wall thrombi and calcifications
- Small native vessel size (below the outer diameter of the used delivery sheath)
- Prominent tortuosity of the iliac arteries and aorta.

#### Endovascular approach

Practically, the minimal luminal (excluding vessel wall) diameter of the access vessels on both sides (common femoral artery, external iliac artery and common iliac artery as well as abdominal and thoracic aorta) should be determined using double-oblique reformations to obtain the correct cross-sectional diameter perpendicular to the longitudinal vessel axis.

Selection of anatomic access point should be based on the concept of selecting the least invasive possible route for TAVI. As previously stated, transfemoral approach should always be the first option, with an alternative approach to be only selected in the setting of a prohibitively luminal compromise due to the mentioned risk factors.

At present, specific cut-offs for the definition of tortuosity degree or extent of calcifications have not been established.

#### Non-endovascular approach

Any potential obstruction or complication along the chosen access route should be reported. For LV transapical access, no abnormalities should be present in the regional thoracic wall or in the apical myocardium (e.g. apical infract with apical thrombus). Also, the angulation between the LV apex and the LVOT must be documented, as steeper angles may complicate the procedure with rigid delivery systems, and is associated with a higher incidence of post-procedural PAR [56, 57].

If a transaortic approach is considered, the amount and location of wall calcification in the ascending aorta should be documented, as extensive anterior aortic wall calcification may compromise device passage and increase the risk of complications. Furthermore, any abnormalities in the adjacent lung parenchyma and thoracic wall should be described.

## Recommended standardised medical report in pre-TAVI assessment

### Consensus statement

- *The report of a pre-TAVI assessment CT or MRI should include all relevant information and measurements of the aortic root and access routes.*
- *Structured reports are highly recommended to ensure all relevant information is included and facilitate communication of results.*

For the analysis of pre-TAVI assessment examinations, structured reports are highly recommended to ensure that all necessary information to TAVI is provided by the report and to assist in consistent communication of data between various sites and specialists involved in the procedural planning.

Furthermore, a standardised structure allows for fast and easy recognition of the relevant findings for the clinician performing the TAVI procedure. Finally, it is a useful educative tool for residents and CT technicians. As a proposal, a template for structural reporting is available on the ESCR website (www.escr.org), accessible for ESCR members only.

In the following, the requested parameters that have to be addressed in a pre-TAVI report are listed and explained.

Since the usual patients evaluated for a TAVI procedure are aged, renal impairment is frequent. Thus, low contrast volumes should be used, which is possible in TAVI evaluation without the risk of insufficient arterial enhancement, since the severe aortic stenosis, per definition present in TAVI patients, leads to reduced cardiac output. The iodine concentration and total contrast volume given should be provided in the standardised report.

The report prior to TAVI should contain measurements and statements about three different anatomical levels: aortic valve and aortic annulus, sinus of Valsalva and access vessels.

For the description of the aortic valve and aortic annulus, a qualitative description of aortic valve calcification should be provided in similarity to the grading that was introduced for echocardiography. Thus, aortic valve calcification should be described as none, mild, moderate and severe. Additionally, asymmetric extension of calcifications either to the aortic-mitral curtain or to the membranous septum should be mentioned, as well as possible morphological abnormalities (for example bicuspid valve). Furthermore, the diameter of the aortic annulus should be measured. Addressing the asymmetric shape of the annulus, a single diameter is not sufficient. Thus, at least the maximal and the minimal diameters of the aortic annulus at systole have to be reported. Alternatively, and more accurately, the area could be measured. The prosthesis size can be inferred from those measurements and suggested to the cardiology team.

The second anatomical target region to be assessed is the sinus of Valsalva. Severely calcified atheromas at the level of the sinus and the ascending aorta have to be described. Although still in most centres invasive coronary angiography is performed in all patients prior to TAVI, coronary arteries should be described in the CT report as well. This information includes the presence or absence of coronary anomalies or anatomical variants of coronary anatomy, as well as the presence or absence of coronary calcifications. To avoid any compromise of coronary ostia by aortic leaflets, the minimal distance between the aortic annulus and the coronary orifices has to be measured and reported. Also, the three diameters—referring to the three parts of the sinus—should be assessed. Additionally, the height of the sinus of Valsalva and the aortic diameter at the level of the STJ and at 40 mm distally to the aortic annulus are important measurements for appropriate planning prior to a TAVI.

The third crucial part of any CT report prior to TAVI consists in the description of the access vessels. The main parameters include vessel tortuosity, vessel wall calcifications and minimal diameter along the access route. Severe thrombotic and/or atherosclerotic wall changes within the entire aorta have to be reported. To avoid any confusion, it is recommended to describe the important findings for each side separately and to end up with a recommendation of the preferred access route. The severity of vessel tortuosity and calcification along the iliaco-femoral axis should be graded for both sides using a three-point qualitative grading score ranging from low to moderate up to severe. Furthermore, the minimal and mean diameters have to be measured at the level of the puncture site (common femoral artery), for the common femoral and external iliac, and for the common iliac artery. Calcified plaques and the presence and severity of stenosis should be documented for the vascular territories described above as well.

A critical and difficult issue for CT assessment prior to TAVI is the fact that a CTA of the entire aorta represents in fact a CT of the whole body. Thus, beside all assessments and measurements as described above, careful assessment of extra-arterial findings is needed—even if it is time-consuming. Given the known comorbidities and the age of the usual TAVI patients, the pre-test probability for unexpected findings potentially important for further patient care and outcome is relatively high. Consequently, every relevant unexpected finding should be reported even during pre-TAVI assessment since this can also influence the treatment decision-making process.

A proposed template for pre-TAVI standardised report is provided in Supplementary Material A.

## Conclusions

Clinical decision-making for the selection of TAVI candidates is a complex multifactorial process taking into account not only specific hemodynamic and anatomic features but also a more general comprehension of the risk-benefit ratio of the procedure, with regard for the patient’s intrinsic frailty and degree of disability [9].

Multimodality imaging plays an important role in this multidisciplinary decision-making, allowing accurate selection of the appropriate valvular device and procedural access, to minimise complications rate and improve the patient’s outcome.

CT remains the preferable and most utilised tool in clinical practice to obtain comprehensive pre-procedural information from annular and aortic root size and morphology to coronary arteries and peripheral vascular anatomy [9].

MR is an alternative modality in case CT is no option, combining accurate morpho-functional assessment of aortic valvular disease with prognostic information derived from the assessment of tissue fibrosis; vascular anatomy can also be assessed using unenhanced free breathing or navigator-assisted techniques which allow to minimise renal exposure to contrast media with impaired renal function [35, 37].

Regardless the method chosen for pre-procedural imaging, rigorous standardisation of the scanning protocols, measurements and medical reporting is critical to assure an adequate image quality and consistency of reported data and terminology between different centres and physicians.

## Electronic supplementary material

ESM 1 (DOCX 41 kb)

ESM 2 (DOCX 29 kb)

ESM 3 (DOCX 26796 kb)

ESM 5 (AVI 49383 kb)

ESM 6 (AVI 19560 kb)

ESM 7 (AVI 32178 kb)

ESM 8 (AVI 15576 kb)

ESM 9 (AVI 20525 kb)

ESM 10 (AVI 50878 kb)

ESM 11 (AVI 21542 kb)

## Abbreviations

AS: Aortic valve stenosis
AVA: Aortic valve opening area
BE: Balloon-expandable valves
CE-MRA: Contrast-enhanced MR angiography
ESCR: European Society of Cardiovascular Radiology
LAO: Left anterior oblique
LVEF: Left ventricle ejection fraction
LVOT: Left ventricular outflow tract
OCA: Ostia of coronary arteries
PAVR: Percutaneous aortic valve replacement
RAO: Right anterior oblique
SE: Self-expandable valves
SOV: Sinuses of Valsalva
SSFP: Steady state free precession
STJ: Sinotubular junction
TAVR or TAVI: Aortic valve replacement or implantation
THV: Transcatheter heart valves
