# Correction to: CT and MR imaging prior to transcatheter aortic valve implantation: standardisation of scanning protocols, measurements and reporting—a consensus document by the European Society of Cardiovascular Radiology (ESCR)

**DOI:** 10.1007/s00330-020-06741-9

**Published:** 2020-03-02

**Authors:** Marco Francone, Ricardo P. J. Budde, Jens Bremerich, Jean Nicolas Dacher, Christian Loewe, Florian Wolf, Luigi Natale, Gianluca Pontone, Alban Redheuil, Rozemarijn Vliegenthart, Kostantin Nikolaou, Matthias Gutberlet, Rodrigo Salgado

**Affiliations:** 1 Department of Radiological, Oncological and Pathological Sciences, Sapienza University, Policlinico Umberto I, V.le Regina Elena 324, 00161 Rome, Italy; 2 Department of Radiology & Nuclear Medicine, Erasmus MC, Rotterdam, The Netherlands; 3 Department of Radiology, University of Basel Hospital, Basel, Switzerland; 4 Department of Radiology, Normandie University, UNIROUEN, INSERM U1096 – Rouen University Hospital, F 76000 Rouen, France; 5 Division of Cardiovascular and Interventional Radiology, Department of Biomedical Imaging and Image-Guided Therapy, Medical University of Vienna, Vienna, Austria; 6 Department of Radiological Sciences - Institute of Radiology, Catholic University of Rome, “A. Gemelli” University Hospital, Rome, Italy; 7 Centro Cardiologico Monzino, IRCCS, Milan, Italy; 8 Institute of Cardiometabolism and Nutrition (ICAN), Paris, France; 9 Department of Cardiovascular and Thoracic, Imaging and Interventional Radiology, Institute of Cardiology, APHP, Pitié-Salpêtrière University Hospital, Paris, France; 10 Laboratoire d’Imagerie Biomédicale, Sorbonne Universités, UPMC Univ Paris 06, INSERM 1146, CNRS, 7371 Paris, France; 11 Department of Radiology, University of Groningen, University Medical Center Groningen, Groningen, Netherlands; 12 Department of Diagnostic and Interventional Radiology, University of Tuebingen, Tübingen, Germany; 13 Diagnostic and Interventional Radiology, University of Leipzig-Heart Center, Leipzig, Germany; 14 Department of Radiology, Antwerp University Hospital, Antwerp, Belgium; 15 Department of Radiology, Holy Heart Hospital, Lier, Belgium

**Correction to: European Radiology**

10.1007/s00330-019-06357-8

The original version of this article, published on 05 September 2019, unfortunately contained a mistake. The image of “Jena Valve” in Table 3 was incorrect. The corrected table is given below.

**Table 3:**
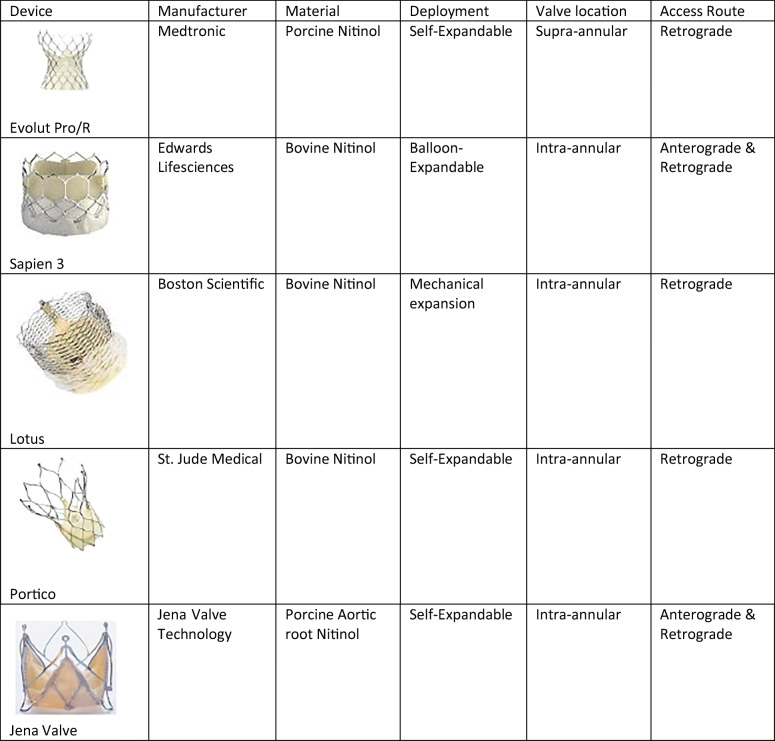
Overview of new generation TAVI devices

