# Supplementary material for: CT and MR imaging prior to transcatheter aortic valve implantation: standardisation of scanning protocols, measurements and reporting—a consensus document by the European Society of Cardiovascular Radiology (ESCR)
Source: Eur Radiol. 2019 Sep 5;30(5):2627–50. doi: 10.1007/s00330-019-06357-8 (PMC7160220; doi:10.1007/s00330-019-06357-8)
Supplement: Supplementary file 2 — (DOCX 41 kb) [file 330_2019_6357_MOESM1_ESM.docx]

Appendix 1.

TAVI CT recommended scan protocols

# General recommendations

- All contrast media with an iodine concentration of at least 300 mg/ml are approved
- Contrast is to be pre-heated at 37 °C
- Administration is through an antecubital vein with preferably an 18 G needle, or 20 G if required
- No B-blockers or other medication should be given
- The amount of iodinated contrast and mA, kV settings should be adapted to the specific scanner type and BMI of the patient for optimal image quality
- Systolic images are preferred for aortic root measurements, although image quality prevails over actual phase of cardiac cycle.
- Image quality should be preferably preserved over 20-80% range of the cardiac cycle
- All measurements should be made using double-oblique reformations perpendicular to the longitudinal axis of the investigated structure or vessel.
- Post-processing should further include:
  - for the aortic root: 10 equally spaced reconstruction phases along the cardiac cycle for optimal evaluation in systolic and diastolic phase, if retrospectively scanned
  - Volume-rendered images and coronal maximum intensity projections of the aorta and iliac arteries for evaluation of tortuosity
  - Curved MPR-evaluation of all involved vessel for luminal patency
  - Workstation-based review of multiplanar reformatted images of the thoracic aorta and chest for other relevant findings

# Single-source 64/128 slice CT scanners

| **Parameter** | **ECG-Gated CTA Aortic root** | **Aorto-iliac access sites** |
| --- | --- | --- |
| Anatomic coverage | Halfway the ascending aorta to above the diaphragm | Above the aortic arch including subclavian artery to below bifurcation of common femoral arteries |
| Scan type | ECG-gated acquisition emphasizing 20-80% of the R-R interval | Non-ECG gated Helical |
| Slice thickness (mm) | ≤1 mm | ≤1.5 mm |
| Tube voltage, kV | 100-120* | 100 |
| mAs per rotation | 500* | 500-700* |
| mA modulation | 20-80% of cardiac cycle | N/A |
| Field of view (mm) | 250, centered on aortic root | 500 |
| Pitch | 0.2–0.24 | >1 |
| Scan direction | Craniocaudal | Craniocaudal |
| Iodinated contrast volume | 90-100 ml*** |  |
| Injection rate | 4.5-5.5 ml/second | N/A, consecutive scan |
| Bolus tracking (threshold start) | At ascending aorta  (HU >100) | N/A, consecutive scan |
| Reconstruction filters | Medium smooth convolution  kernel | Medium smooth convolution  kernel |
| Iterative reconstruction | Yes, medium strength | Yes, medium strength |

* Following adaptations are suggested according to BMI

| BMI | kV | mA range |
| --- | --- | --- |
| <20 | 100 | 100-400 |
| 20-25 | 120 | 250-550 |
| 25-29 | 120 | 300-650 |
| >=30 | 140 | 300-750 |

# Dual-source CT scanners, non-high pitch mode

| **Parameter** | **ECG-Gated CTA Thorax**  **Heart rate > 60 bpm** | **Aorto-iliac CTA Below Diaphragm** |
| --- | --- | --- |
| Anatomic coverage | Whole aortic arch down to cardiac apex  Extension to entire subclavian arteries if possible access route option | Above the aortic arch including subclavian artery to below bifurcation of common femoral arteries |
| Scan type | Retrospective ECG-gating | Non-gated helical |
| Collimation, mm | 2 × 32 × 0.6 | 32 × 0.6 |
| Slice thickness, mm | ≤1 mm | 1-3 mm |
| Tube voltage, kV | 100-120* | 120 |
| Tube current–time product, mAs per rotation | 320 160 | 240 |
| Field of view (mm) | 250 for aortic root evaluation | 500 |
| Pitch | 0.2–0.43** | 0.7 |
| Scan direction | Craniocaudal | Craniocaudal |
| Iodinated contrast volume | 90-100 ml*** |  |
| Injection rate | 4.5-5.5 ml/second |  |
| Bolus tracking (threshold start) | At ascending aorta  (HU >100) | N/A, consecutive scan |
| Reconstruction filters | Medium smooth convolution  kernel with either filtered back projection or iterative reconstruction | Medium smooth convolution  kernel with either filtered back projection or iterative reconstruction |

* 100 kVp: BMI <= 25; 120 kVp: BMI >= 25

** consider ECG-triggered high-pitch spiral acquisition if available, to reduce both contrast and radiation dose; aortic root is usually well assessed even in higher heart rates.

*** Following formula could be applied: Total Volume [in ml] of contrast agent to be injected = (Expected scan time given by scanner [in sec] + 10 [constant]) X Flow of contrast agent [in ml/sec]

# Dual-source CT scanners, high-pitch mode

| **Parameter** | **ECG-Gated CTA Aortic root*** | **High-pitch CTA of the thorax, abdomen and pelvis*** |
| --- | --- | --- |
| Anatomic Coverage | Middle aortic arch to bottom of the heart | Above the aortic arch including subclavian artery to below bifurcation of common femoral arteries |
| ECG gating | Prospective ECG-triggering 30-50% R-R interval | High pitch spiral |
| Collimation, mm | -- x 0.6 mm  (Adapted to fit scan length) | 2 × 128 × 0.6 mm (FLASH)  2 × 192 × 0.6 mm (FORCE) |
| Slice thickness, mm | ≤1 mm | ≤1 mm |
| Tube voltage, kV | Ref. kVp, 120 kVp** | Ref. kVp, 120 kVp** |
| Tube current–time product, mAs per rotation | Reference tube current, 190 mAs/rot*** | Reference tube current, 120 mAs/rot*** |
| Field of view (mm) | 250 | 250 for aortic root evaluation  500 for peripheral vessels |
| Pitch | Not applicable | 1.55 or 3.2-3.4**** |
| Scan direction | Craniocaudal | Craniocaudal |
| Iodinated contrast volume | 70 ml | 40-60 ml*** |
| Injection rate | 45ml@3.0/s contrast  25ml@2.5/s contrast  5ml@2.5/s saline | *4-5.5 ml/second****** |
| Bolus tracking (treshold start) | At ascending aorta  (HU >100) | At ascending aorta  (HU >100) |
| Reconstruction filters | Medium smooth convolution  kernel with either filtered back projection or iterative reconstruction | Medium smooth convolution  kernel with either filtered back projection or iterative reconstruction |

- It is advised to perform a scan with 2 separate acquisitions during the same session. The first to assess the aortic root (ECG-gated CTA aortic root) and the second for the vascular access (high-pitch CTA of the thorax, abdomen and pelvis). The second scan can immediately be started after the first using only the contrast injection as specified for the first scan. Alternatively, only a single acquisition consisting of a high-pitch CTA of the thorax, abdomen and pelvis can be performed, from which the aortic root can be reconstructed. If this is done the contrast injection protocol as specified in italics should be used.

**CARE KV: on (dose saving optimised for ‘vasculature’)

***Anatomical dose modulation: CARE Dose 4D on

**** Depending on field of view size needed

***** Following formula could be applied: Total Volume [in ml] of contrast agent to be injected = (Expected scan time given by scanner [in sec] + 10 [constant]) X Flow of contrast agent [in ml/sec]. Also see remarks under *.

# Wide detector volume CT scanners

| \| **Parameter** \| **ECG-Gated Volume scan of the heart** \| **Access sites from subclavian**  **to femoral level** \| \| --- \| --- \| --- \| \| Anatomic coverage \| Heart & aortic arch \| Above the aortic arch including subclavian artery to below bifurcation of common femoral arteries \| \| Scan type \| Volume scan, ECG-gated, complete heart cycle \| Non-gated helical \| \| Collimation, mm \| 320 x 0.5 \| 80 × 0.5 \| \| Slice thickness, mm \| 0.5 \| 0.5 \| \| Tube voltage, kV \| 100  120 if more than 700 mA \| 100 \| \| Sure exposure (mAs) \| SD 17.5 \| SD 17.5 \| \| Rotation time \| 0.275 \| 0.275 \| \| Pitch \| - \| Standard \| \| Field of view \| Body std Volume 1/1 \| Body std Volume 1/1 \| \| Scan direction \| Craniocaudal \| Craniocaudal \| \| Iodinated contrast volume \| 90-100 ml*** \| N/A, consecutive scan \| \| Injection rate (@ >350 mg Iodine/ml) \| < 75 kg: 70 ml @ 4,0 ml/s   - 1. kg: 80 ml @ 4,5 ml/s   > 110 kg: 90 ml @5 ml/s \| \| Bolus tracking \| Surestart with ROI in descending aorta in the middle of the volume \| \| Reconstruction filters \| FC03: Cardiac CTA std 0.5/0.25  Iterative reconstruction: AIDR3D enhanced \| FC03: Cardiac CTA std 1.0/0.8  Iterative reconstruction: AIDR3D enhanced \| \|  \|  \|  \| |
| --- | --- | --- | --- | --- | --- | --- | --- | --- | --- | --- | --- | --- | --- | --- | --- | --- | --- | --- | --- | --- | --- | --- | --- | --- | --- | --- | --- | --- | --- | --- | --- | --- | --- | --- | --- | --- | --- | --- | --- | --- | --- | --- | --- | --- | --- | --- |
