# Supplementary material for: CT and MR imaging prior to transcatheter aortic valve implantation: standardisation of scanning protocols, measurements and reporting—a consensus document by the European Society of Cardiovascular Radiology (ESCR)
Source: Eur Radiol. 2019 Sep 5;30(5):2627–50. doi: 10.1007/s00330-019-06357-8 (PMC7160220; doi:10.1007/s00330-019-06357-8)
Supplement: Supplementary file 3 — (DOCX 29 kb) [file 330_2019_6357_MOESM2_ESM.docx]

# Appendix 2.

TAVI MR recommended scan protocol

# 2.1 General recommendations

- An MR examination should only be considered in the presence of contra-indications for CT
- Any system with at least 1.5 T field strength is adequate
- A non-contrast enhanced CT examination can additionally be performed for better visualization of valve and aorto-iliac calcifications
- Administration of contrast medium is through an antecubital vein
- No B-blockers or other medication should be given
- Systolic measurements are preferred, although image quality prevails over actual phase of cardiac cycle.
- All measurements should be made using double-oblique reformations perpendicular to the longitudinal axis of the investigated structure or vessel if possible.
- All breath-hold images are acquired in end-expiration

# 2.2 MR protocol for aortic root & access route

| **Parameter** | **Purpose** | **Comment** |
| --- | --- | --- |
| Localizers centered on thoracic aorta and aortic valve plane | Determination of annular plane  & aortic position |  |
| Black blood ECG-gated half-Fourier FSE images | General chest evaluation | Non-gated axial SSFP* images can also be considered |
| SSFP cine images along 2/3/4-chamber long- and short axis according to guidelines | Chamber evaluation | Short-axis stack needed for calculation of ejection fraction, ventricular volumes and mass |
| Double-oblique long- and short axis SSFP images through the aortic root | Coverage of the aortic root, annular & sinus measurements, height coronary ostia | Breath-hold or 3D Navigator assisted acquisition |
| White or black blood ECG-gated half-Fourier FSE images | Annular & sinus measurements, height coronary ostia | Depending on quality of ECG-gated SSFP acquisition |
| Phase-contrast images for Velocity mapping | Calculation of transvalvular flow velocity, volume and pressure gradient | Consider calcium quantification with CT in extensive valve calcification with inconclusive results |
| Contrast-enhanced 3D GE T1-weighted MR angiography of aorta & iliac vessels | Investigation of luminal patency of access arteries | Two acquisitions are often necessary to cover anatomy from thoracic aorta to below femoral bifurcation  Sequence is not suitable for aortic root measurements  Visual start of sequence using monitoring slice in the ascending aorta |
| Delayed enhancement short and long-axis IR T1-weighted images | Detection of myocardial fibrosis | Also consider T1-mapping techniques for interstitial fibrosis if available |
