# Supplementary material for: CT and MR imaging prior to transcatheter aortic valve implantation: standardisation of scanning protocols, measurements and reporting—a consensus document by the European Society of Cardiovascular Radiology (ESCR)
Source: Eur Radiol. 2019 Sep 5;30(5):2627–50. doi: 10.1007/s00330-019-06357-8 (PMC7160220; doi:10.1007/s00330-019-06357-8)
Supplement: Supplementary file 4 — (DOCX 26796 kb) [file 330_2019_6357_MOESM3_ESM.docx]

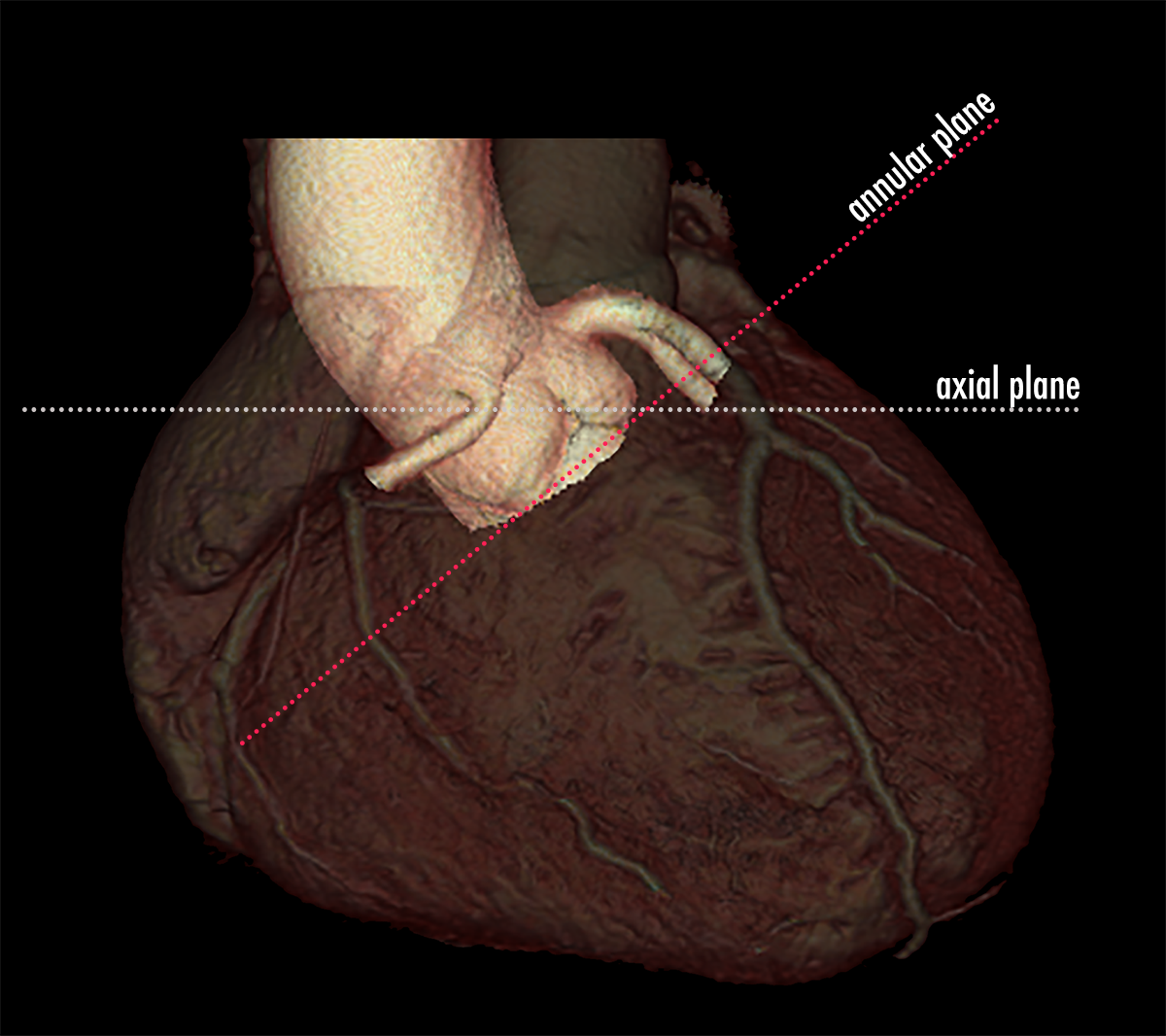


Figure 2

Fig. 2 3D Volume rendering CT image of the heart containing the aortic root. The aortic root has a double-oblique orientation within the heart. Therefore, standard orthogonal imaging planes, like the axial plane indicated with the dotted white line, are not suitable to correctly visualize the aortic root and containing structures. For this reason, intrinsic 3D imaging modalities like CT are necessary to correctly assess the aortic root and annulus and obtain accurate measurements.


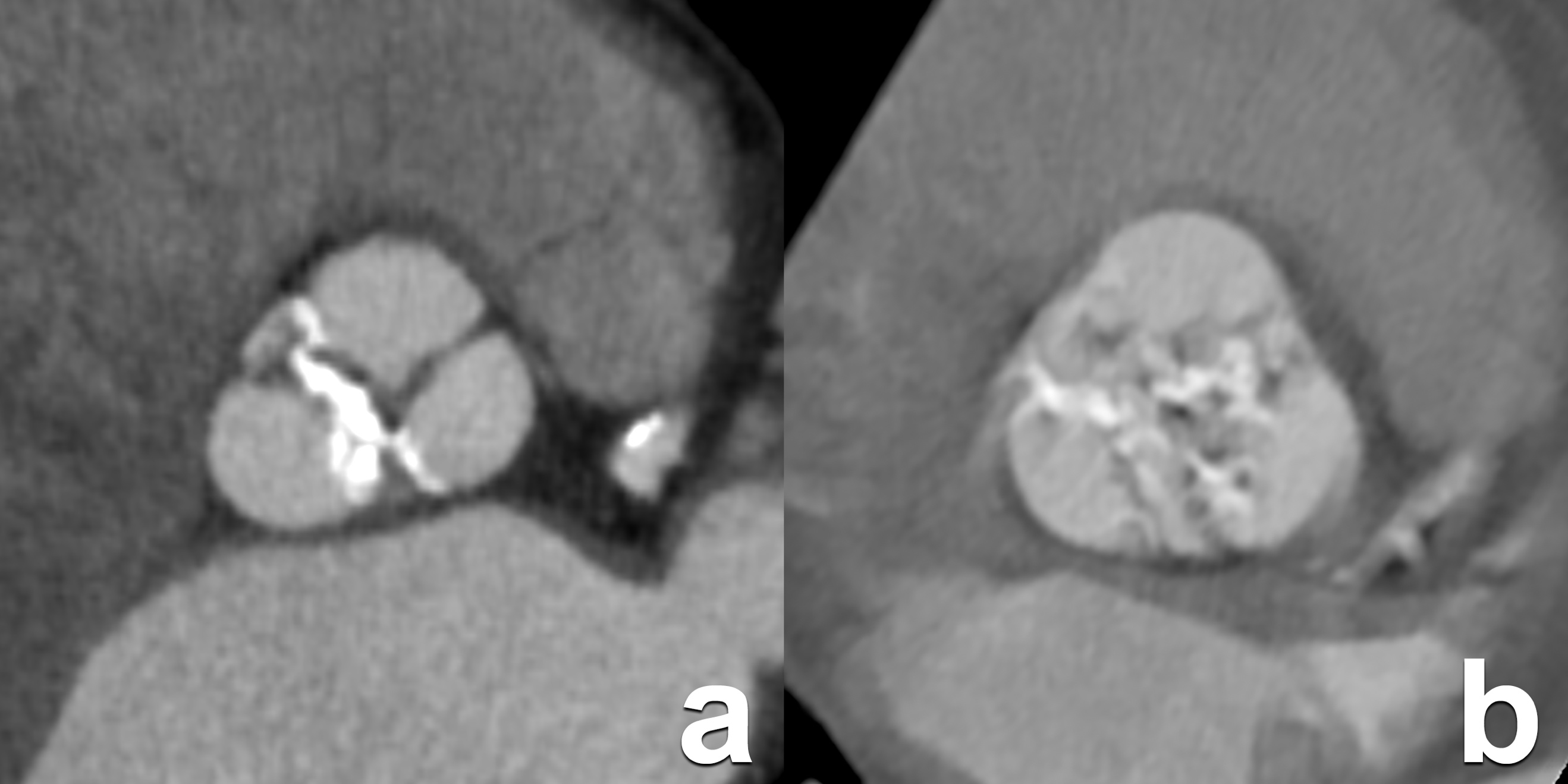


Figure 4

Fig. 4. Most TAVI candidates will present with an aortic valve containing significantly calcified valve leaflets. The majority of patients will have a clearly identifiable tricuspid aortic valve (a). However, a significant portion will have a bicuspid aortic valve, which is an important feature to report as its presence is associated with some specific complications. However, in some cases valve cuspidity can be difficult to assess in heavily degenerated valves, where extensive calcification can make differentiation between tricuspid and (functionally) bicuspid valves difficult (b).


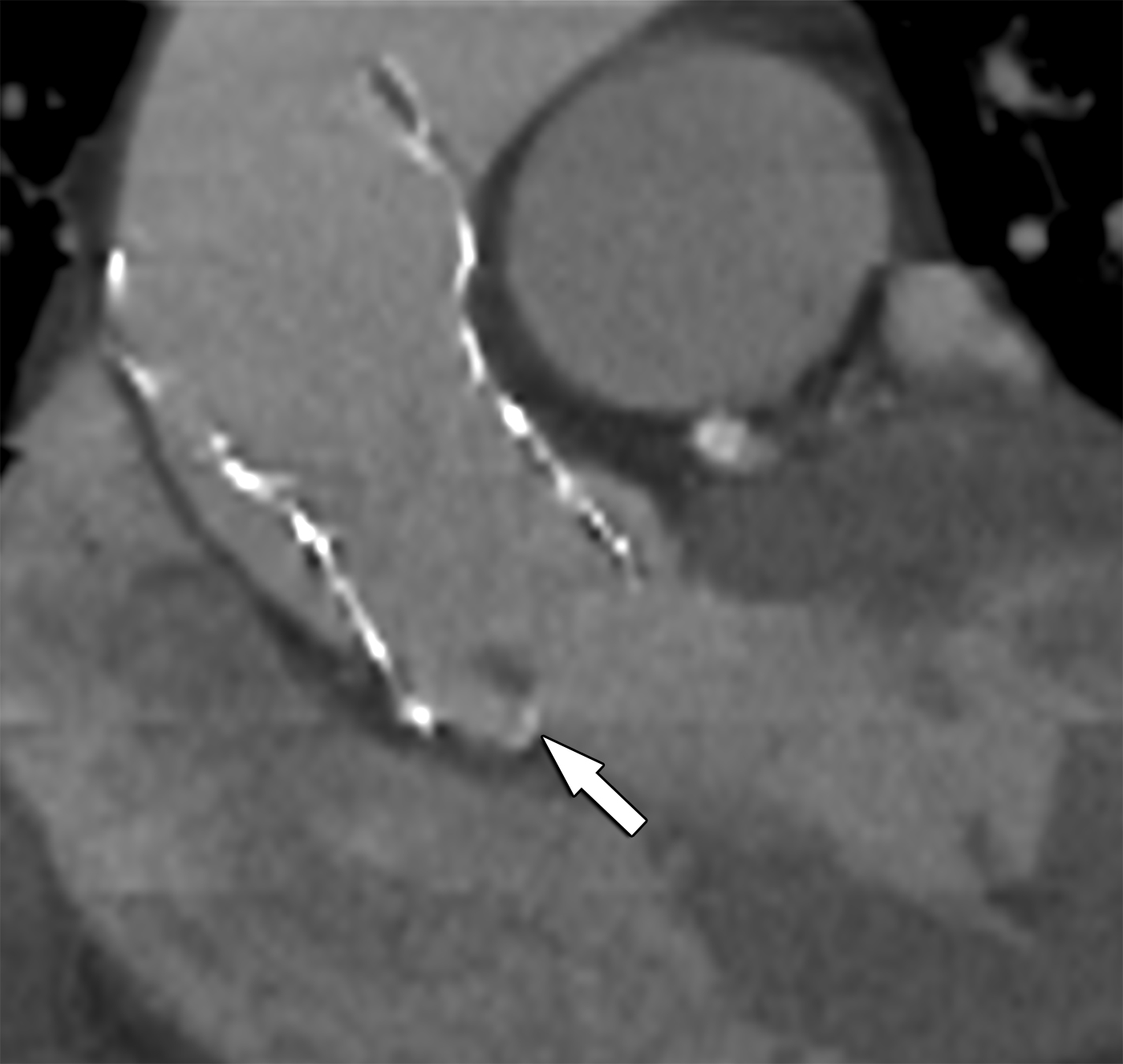


Figure 6

Fig. 6 Incorrect positioned THV, which is tilted and does not fully extend into the aortic annulus. As such, parts of the native right aortic valve leaflet is protruding into the inflow part of this self-expandable THV (arrow), causing a residual valve gradient on Doppler echocardiography. CT is very useful in detecting the cause of THV dysfunction in cases where Doppler echocardiography does not provide an answer. In this case, function was improved after balloon dilatation of the inflow part of the THV, further crushing the remaining valve leaflets against the adjacent aortic wall.


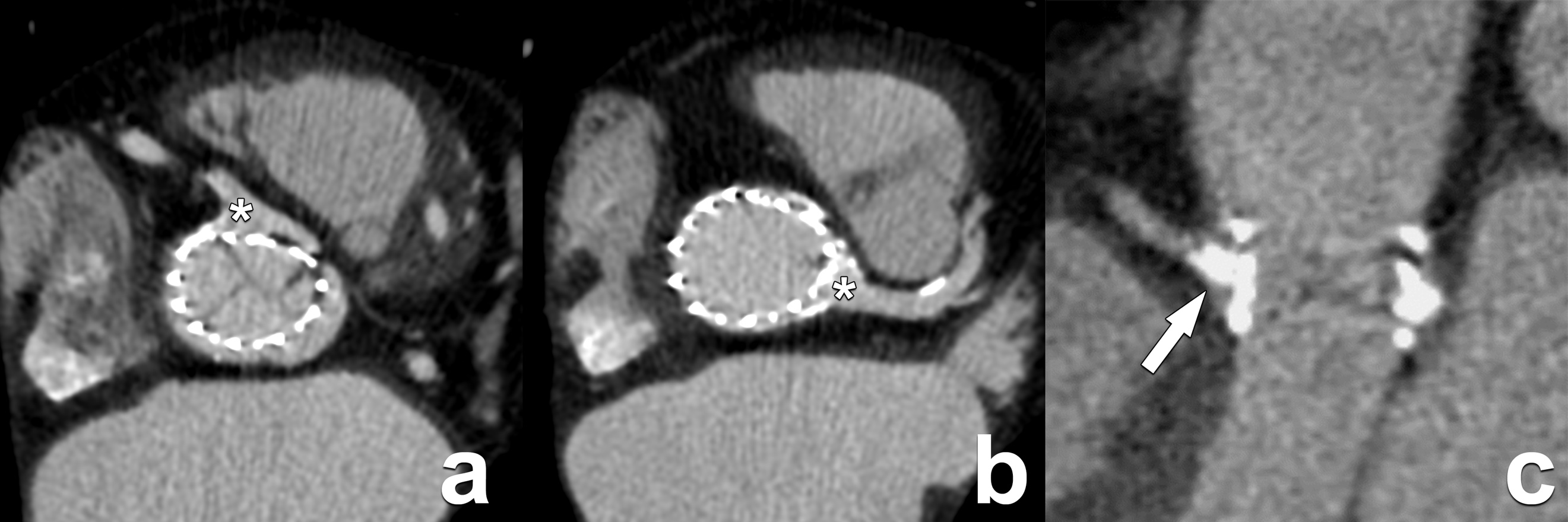


Figure 7

Fig. 7 Relation between the coronary ostia and the deployed THV. Both self-expandable and balloon-expandible THV are designed not to obstruct the coronary ostia, with self-expandble Corevalve and Evolut protheses extending into the ascending aorta by design, leaving the coronary ostia open (a, b). When coronary obstruction occurs, it is not by the THV but secondary to displaced calcified native leaflet remnants that migrate during deployment of the THV in the aortic sinus to the vicinity of the coronary ostia. Nevertheless, while CT can detect these migrated calcifications in or near the coronary ostia (c), the evaluation of luminal patency is less obvious, mostly dependent on local expertise and the quality of the CT scanner used.


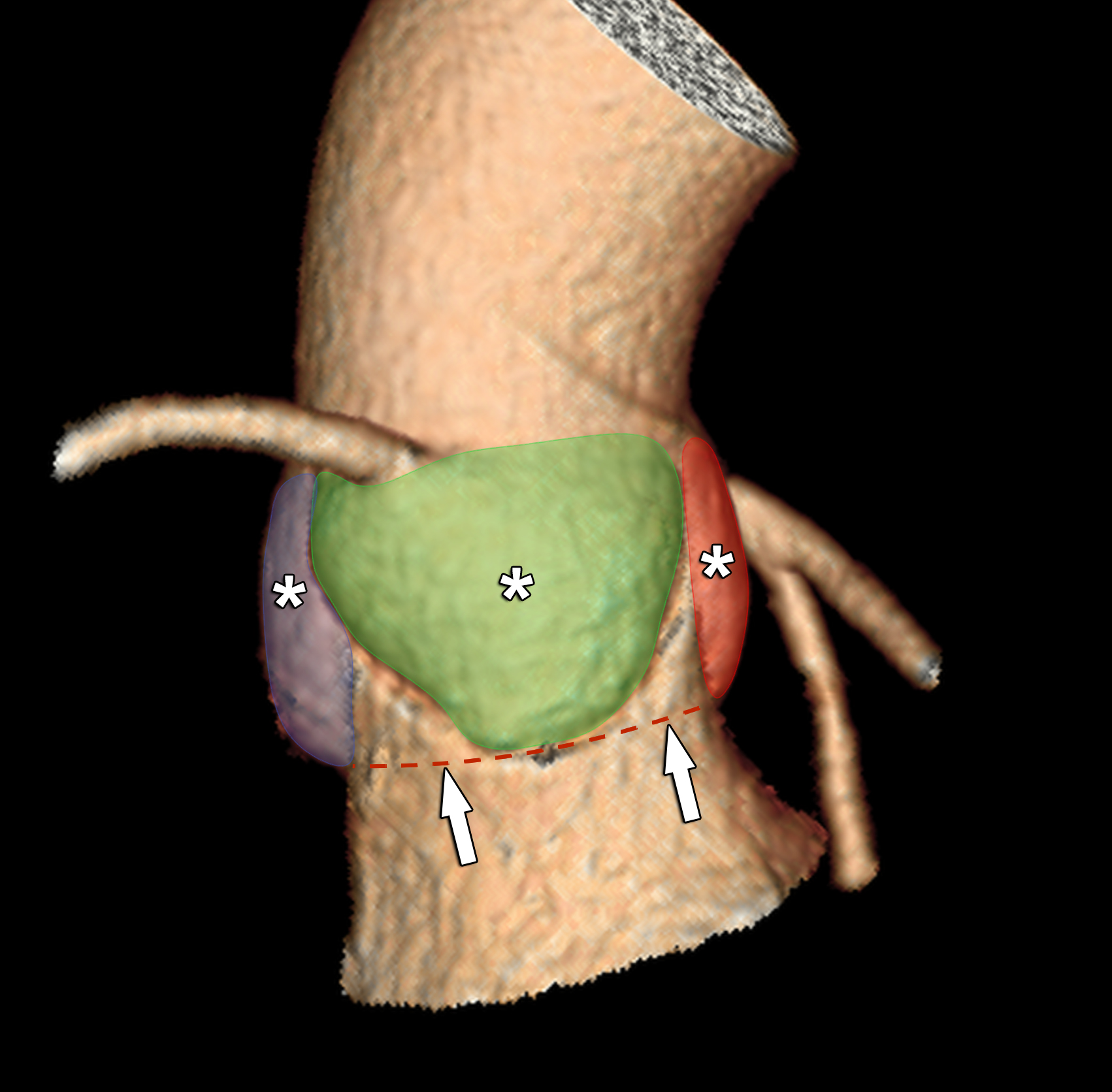


Figure 10

Fig. 10 3D CT image of the aortic root containing the sinuses of Valsalva (asterisk). As the aortic valve leaflets extend within these sinuses up to the sino-tubular junction, connecting their most basal attachment sites forms a virtual ring which is named the aortic annulus (red dotted line, arrows). It also marks the transition to the LVOT.


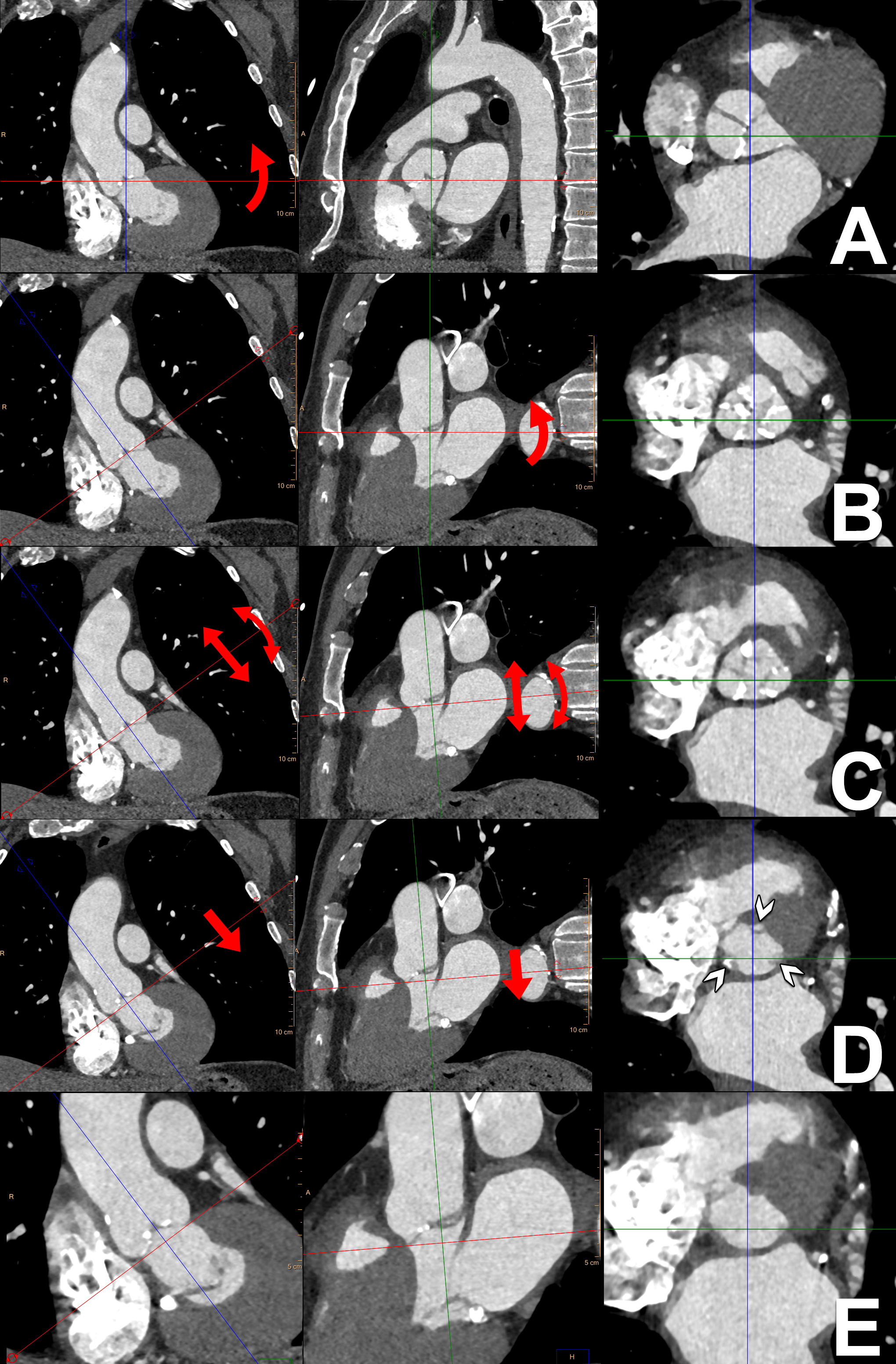


Figure 12

Fig. 12 For all measurements of the aortic root as illustrated in figures 12, 13, and 14 the use of a (simple) multiplanar reconstruction viewer is mandatory. The three imaging planes should be perpendicular to each other at 90-degree angles and the reference lines should be “locked” so rotating one reference line automatically rotates the other planes. Care should be taken to have the screen layout setting in such a way that all three imaging planes (starting with axial, coronal and sagittal) are visible simultaneously.

The CTA dataset (preferably a systolic phase) is loaded into the viewer. A) First, in the coronal plane the aortic valve is located and the centre of the reference lines is placed approximately at the center of the aortic valve. In the coronal image plane, the references lines are rotated so one of the two lines is at approximately 45-degrees to the horizontal level. This results in the images seen in B. In the plane that was the original sagittal reconstruction (middle panel in B) the reference line is also rotated to be approximately parallel to the aortic valve. This generally provides a pretty good imaging plane that is perpendicular to the aortic valve (right panel in B). The essential step (illustrated in C) is to scroll up and down through this image stack (as indicated by the straight arrows in the other views in C) and determine if all three aortic valve cusps are seen symmetrically in each image (i.e. scrolling from the level of the LVOT to the aortic valve the three cusps should appear symmetrically and simultaneously in one image). This is often not yet the case. By tweaking the angulation of the plane by slight rotation of the crosshairs in the other views (as indicated by the curved arrows in C) while assessing its effect on the symmetry of the valve leaflets in the in-plane image is needed to have the cusps appear symmetrically. Once this has been established, by scrolling through the image stack in-plane with the aortic valve towards the LVOT, the leaflets will increasingly appear smaller and closer to the aortic wall (D, see arrowheads in right panel). The first image just below the level of the lowest image (i.e. closest to the LVOT) that no longer shows the leaflets is selected and represents the annulus (E).


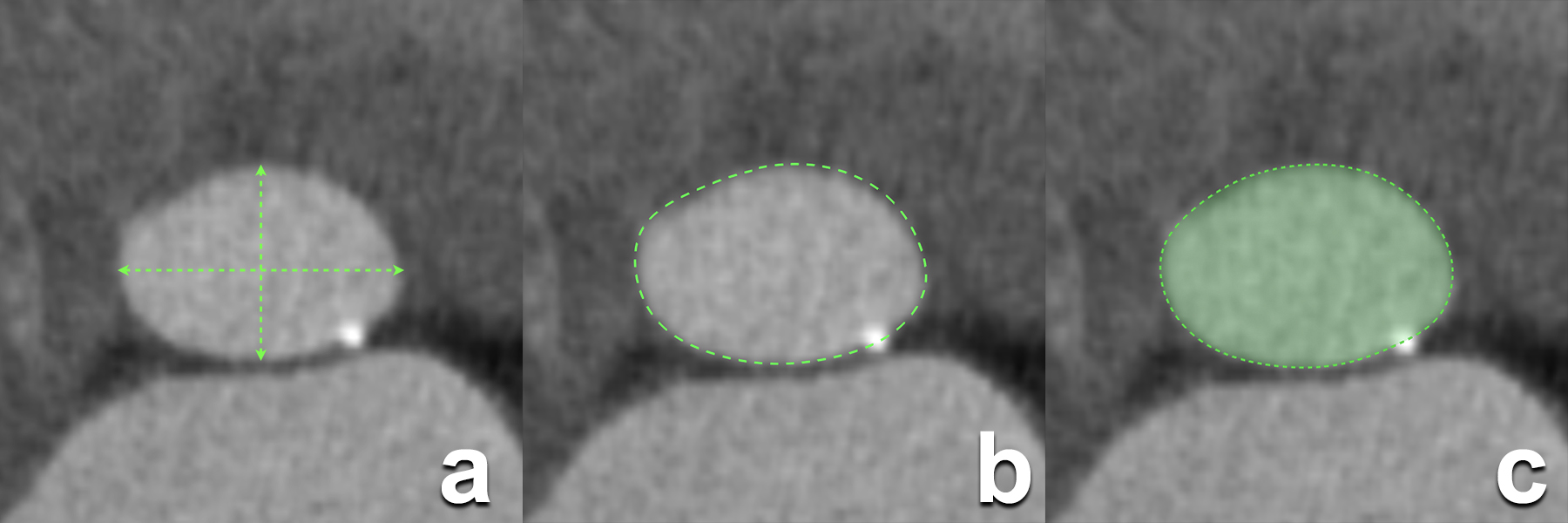


Figure 13

Fig. 13 The annular plane image obtained through the steps outlined in figure 12 is used for the measurements. The long and short axis diameter are measured using a simple distance tool (a). In the annular plane the circumference of the annulus is traced using a planimetry tool (b). Most software systems then automatically display the area, perimeter and area derived diameter of the traced area (c). Alternatively, parameters can be calculated as described in the text.


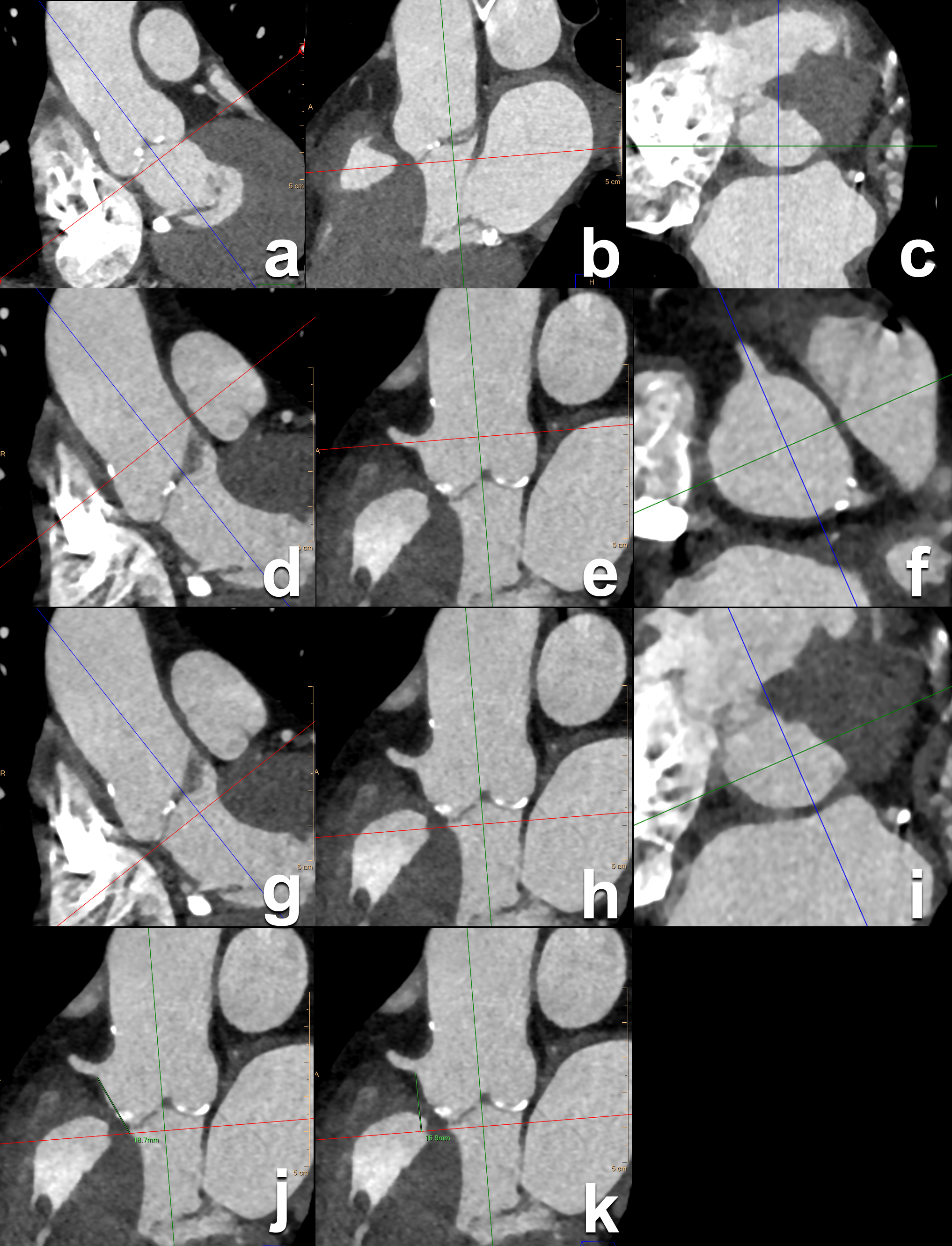


Figure 14

Fig. 14 Standardized way to perform measurements of the distance of the annular plane to the ostium of the right coronary artery and left main. The starting point are the annular plane images (obtained through the steps outlined in Figure 12) as displayed in A-C above. In this image stack, in plane with the annulus, the origin of the RCA is located by scrolling through the images in the direction of the aorta (arrows in A, B) . Subsequently, the reference lines are rotated (curved arrow in C) in such a way that one of the refences lines passes through the RCA ostium (asterix in F); then, by scrolling toward the LVOT (arrows in D, E) the annulus plane is again displayed (I) and in one of the two other panels (H) the origin of the RCA is visible (asterix in H), as well as the reference line which corresponds to the annulus plane level (red line in H). The distance is measured as the distance of the lower border of the RCA ostium (asterisk in J, K) to the attachment of the coronary cusp (J) or perpendicular to the reference line of the annulus plane (K). For the distance of the annulus to the left main, the steps mentioned above are repeated.


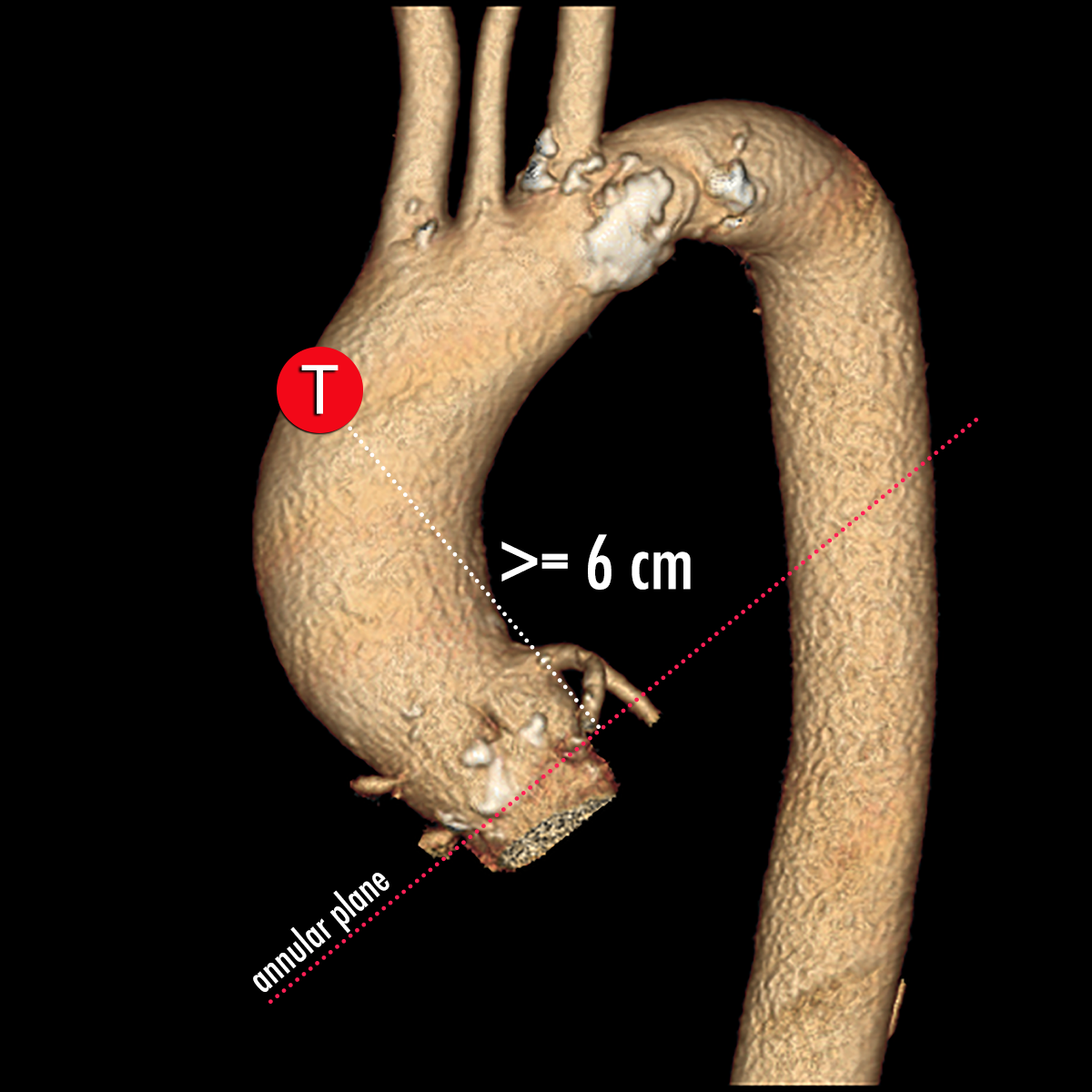


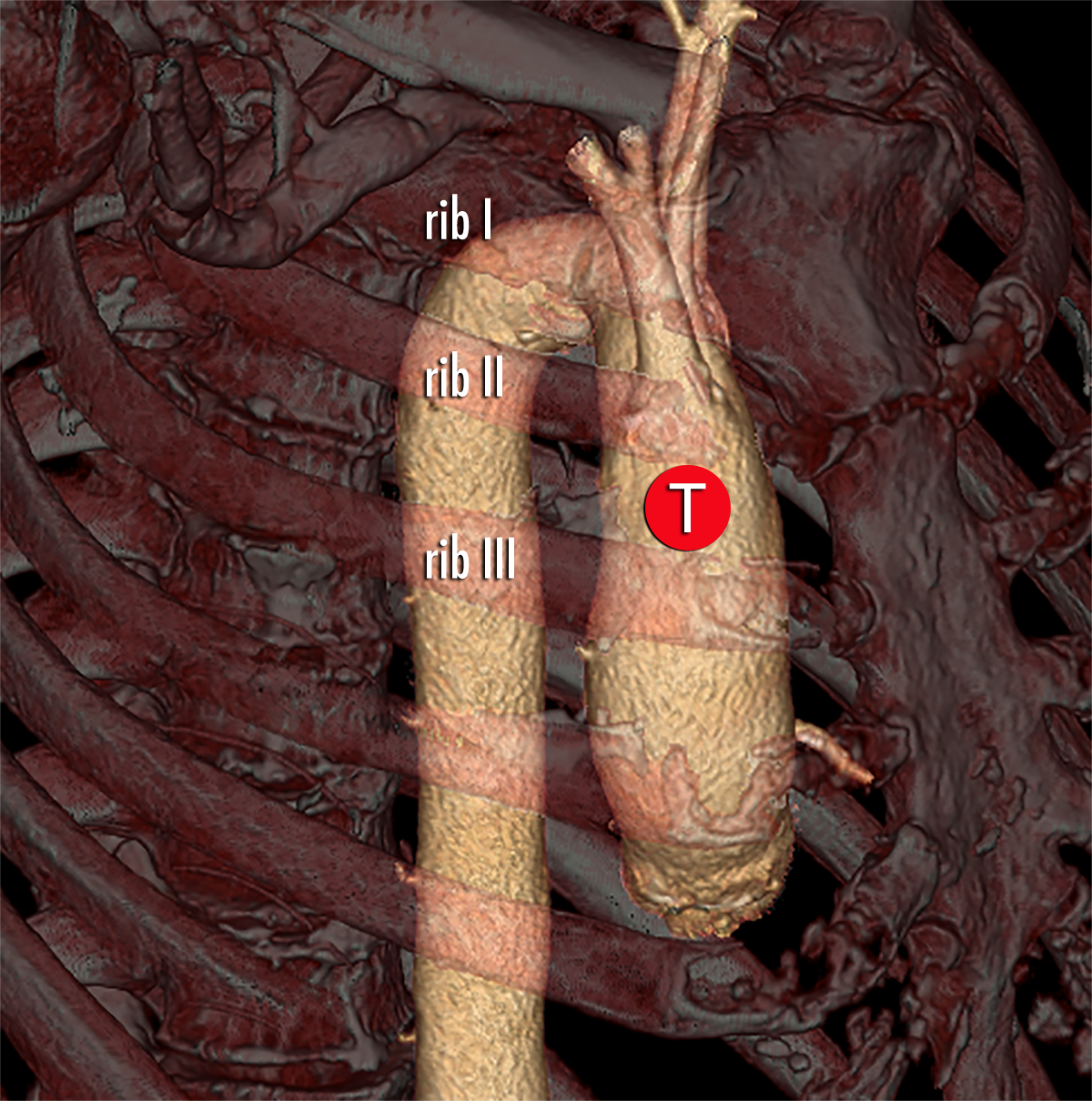


Figure 15 a Figure 15 b


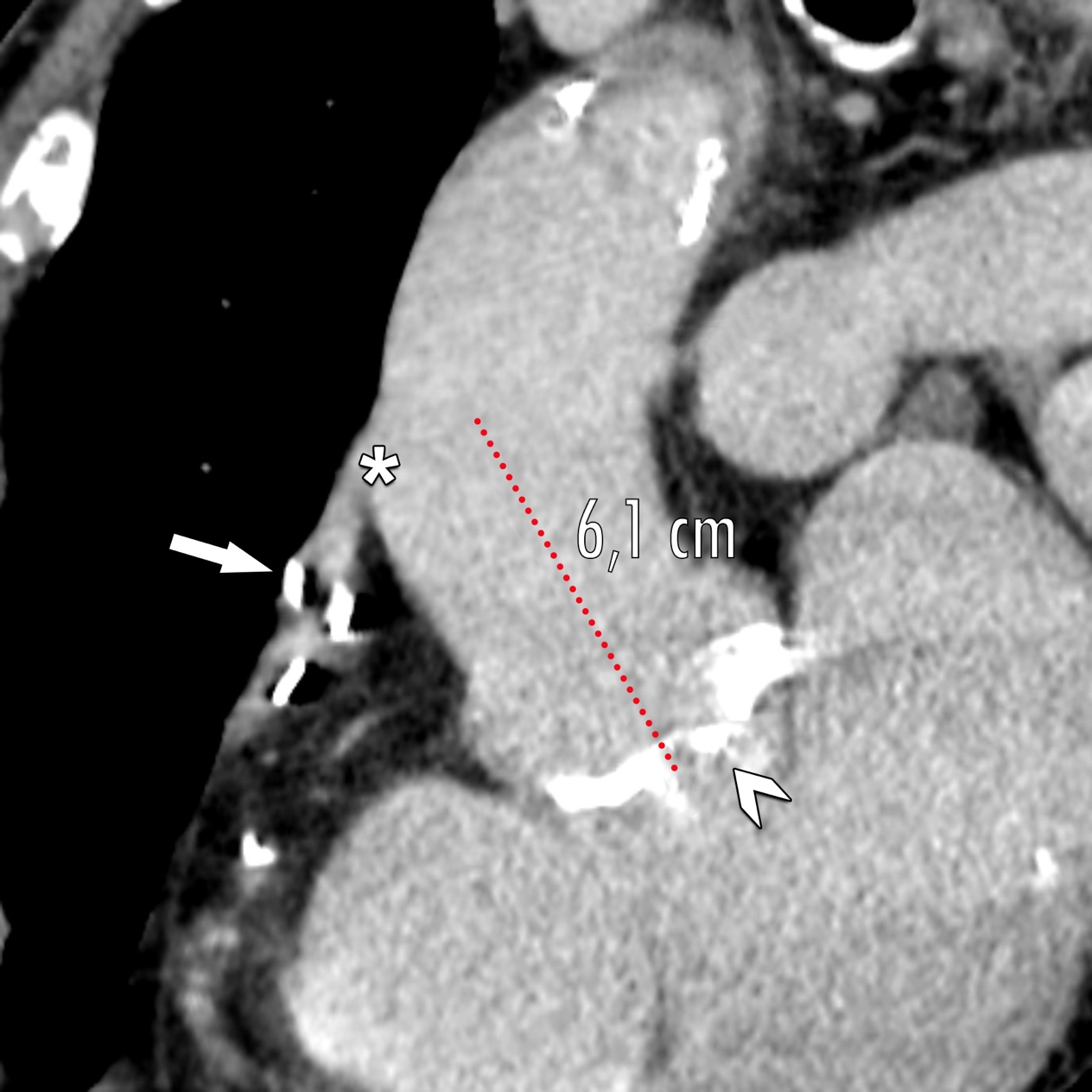

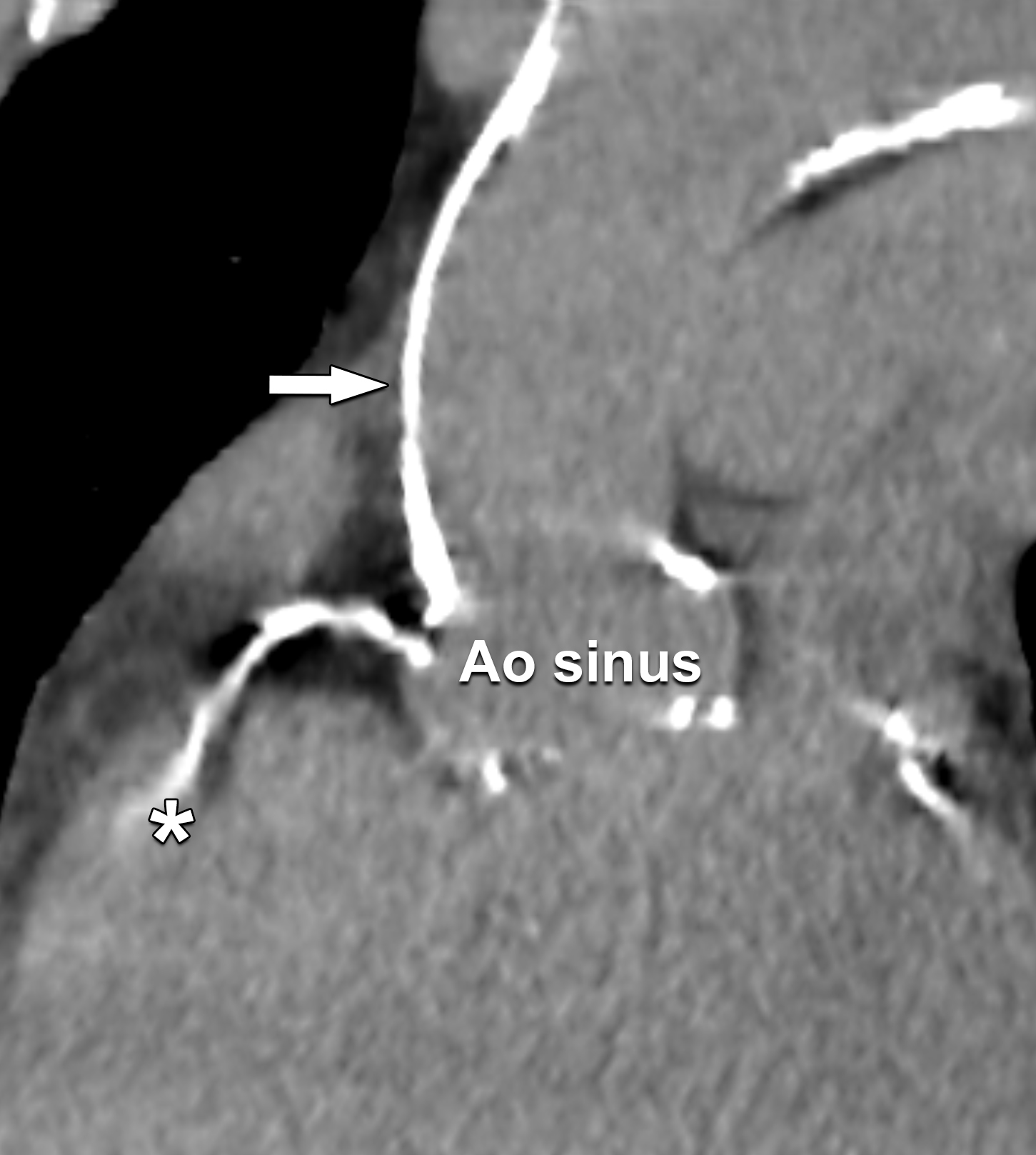


Figure 15 c Figure 15 d

Fig. 15 Illustration of the target point (T) in case of a transaortic THV delivery. When the usual endovascular or transapical delivery routes are not possible, the transaortic pathway offers an alternative for patients in which no other access is possible. The recommended entry point for self-expandable THV is located at least 6 cm above the level of the aortic annulus (annular plane in red dotted line). Furthermore, the status of the aortic wall around this location needs to be scrutinized, as e.g. extensive calcification increases procedural feasibility and risk, and may as such make this access path unsuitable.

However, even for transaortic access caution is needed. The TAVI candidate might have previous coronary bypass grafts, including a venous bypass over the RCA (arrow in c). The origin of this bypass (asterisk in c) might be near the targeted entry point, in this case about 6 cm above an annular plane with a heavily calcified aortic valve (arrowhead in c). Also, the anterior wall of the ascending aorta may show extensive calcification (arrow in d), making transaortic access impossible. Note incidental visualisation of a extensive calcified RCA (asterisk in d).


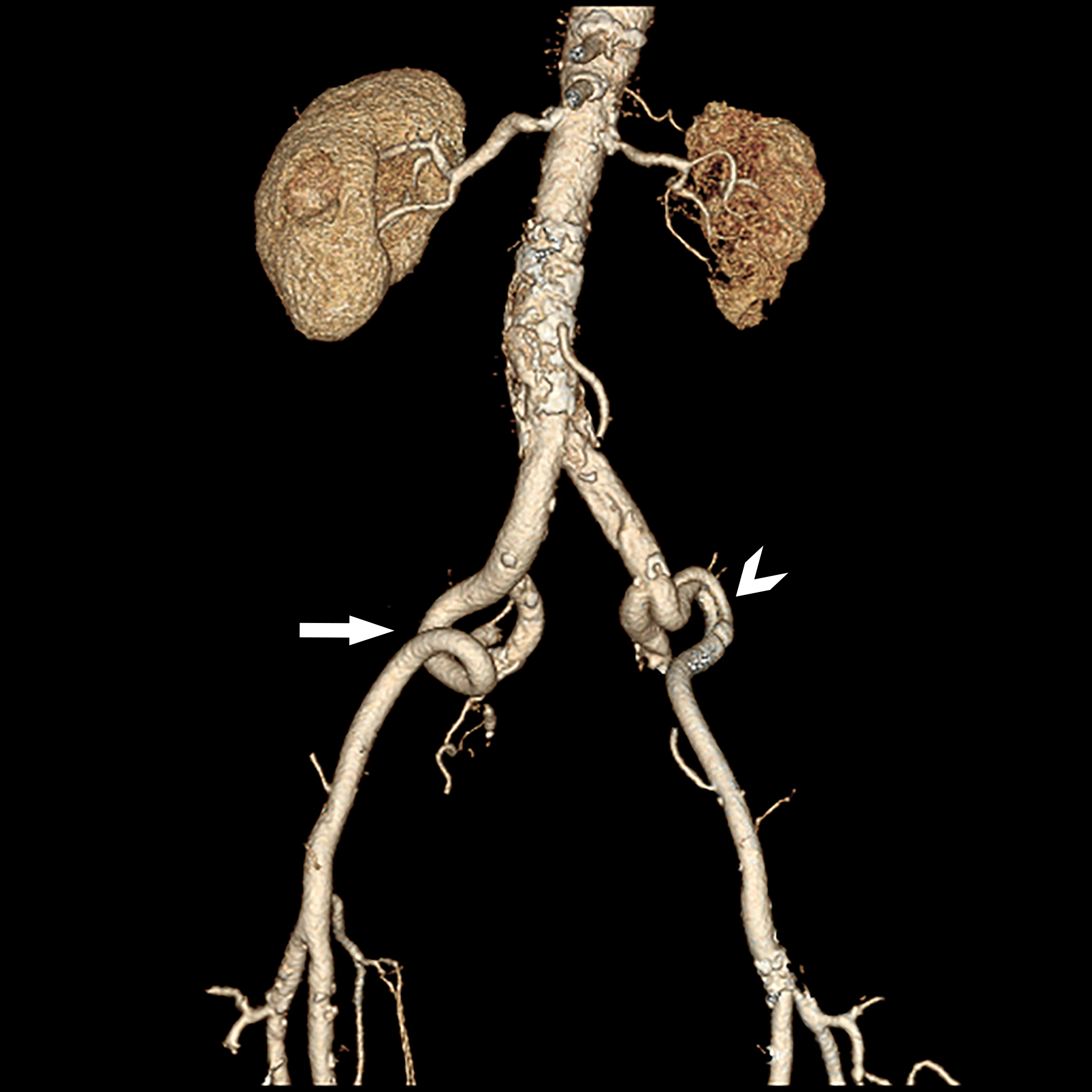


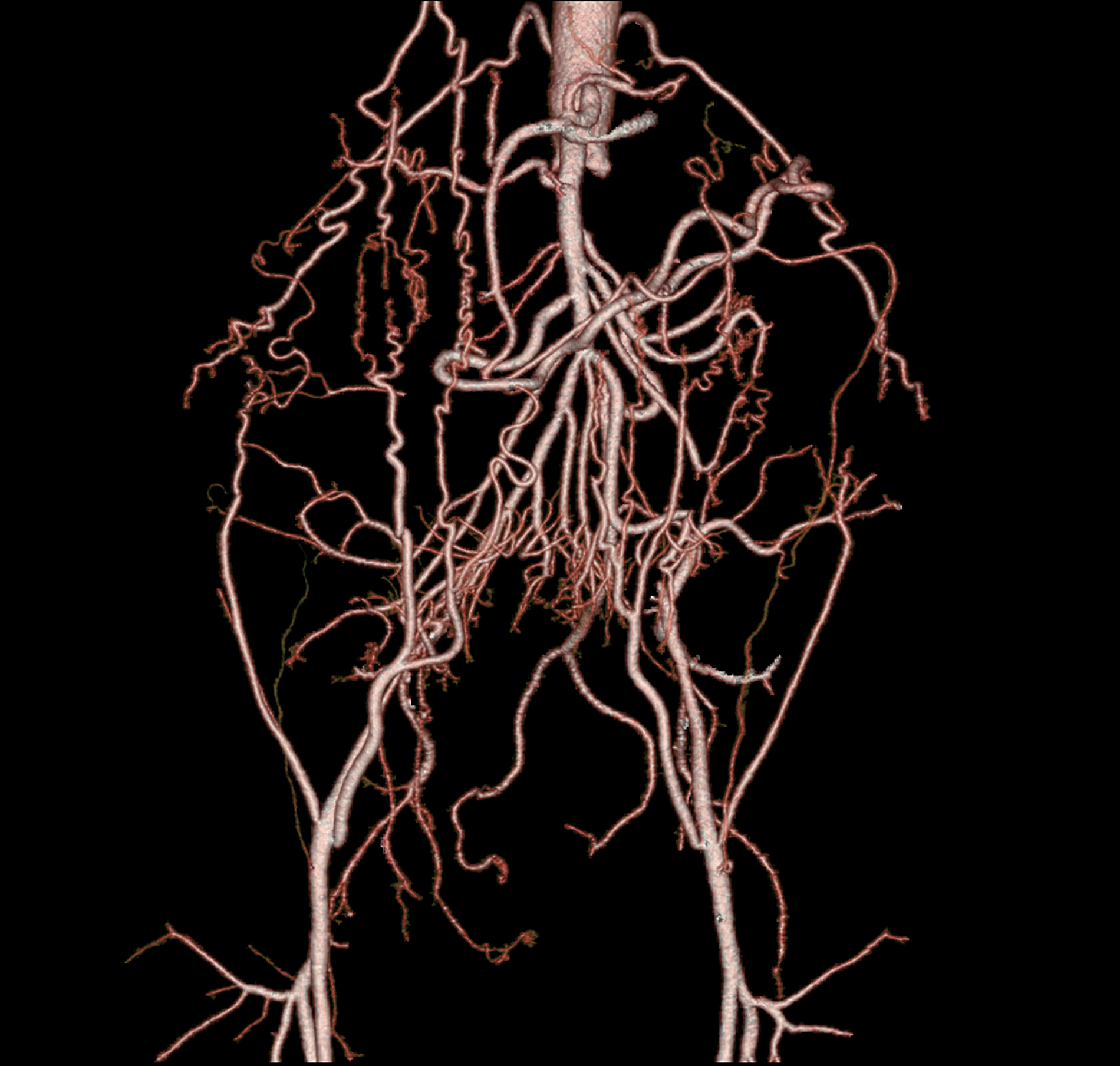


Figure 16 a Figure 16 b


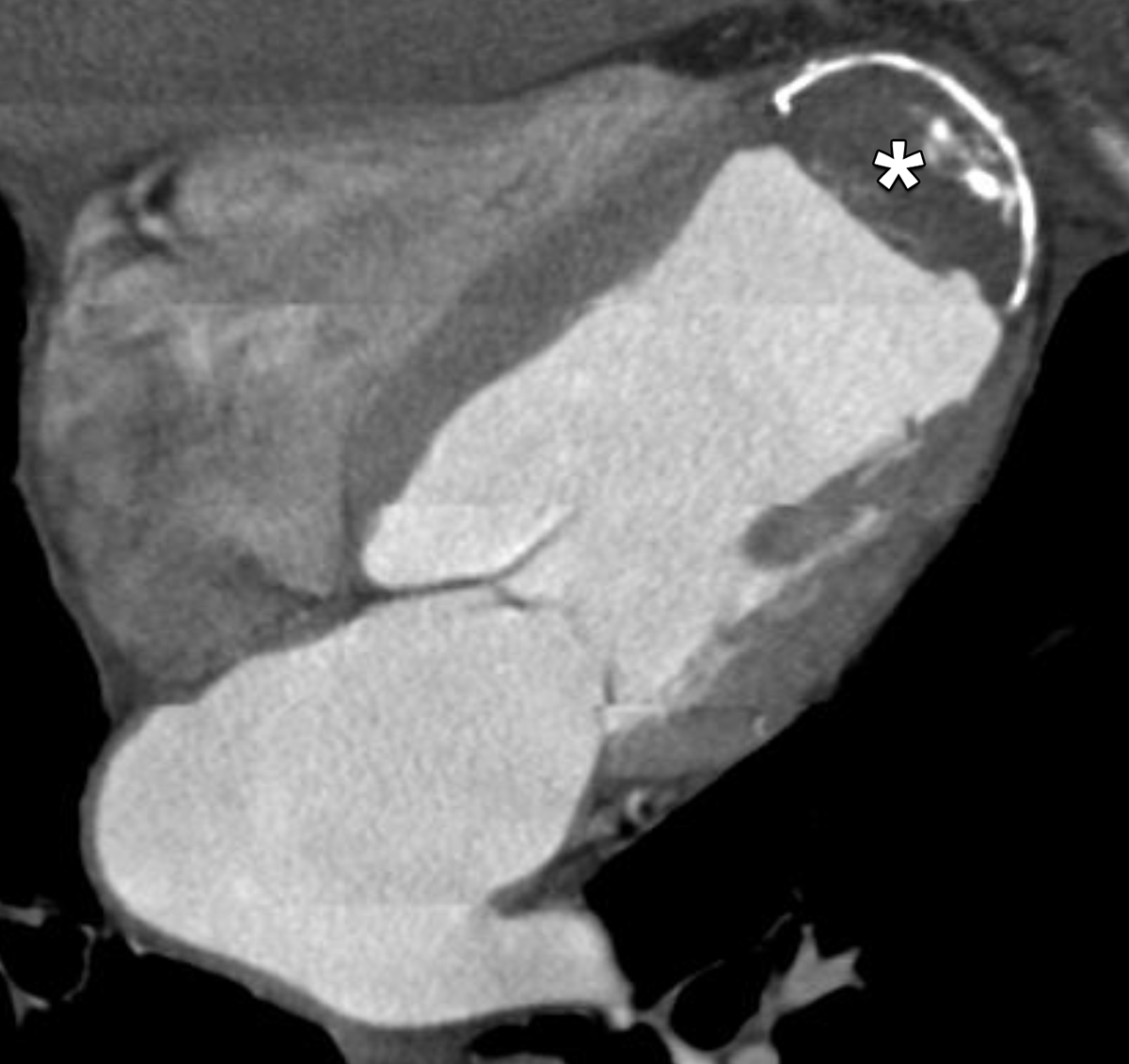

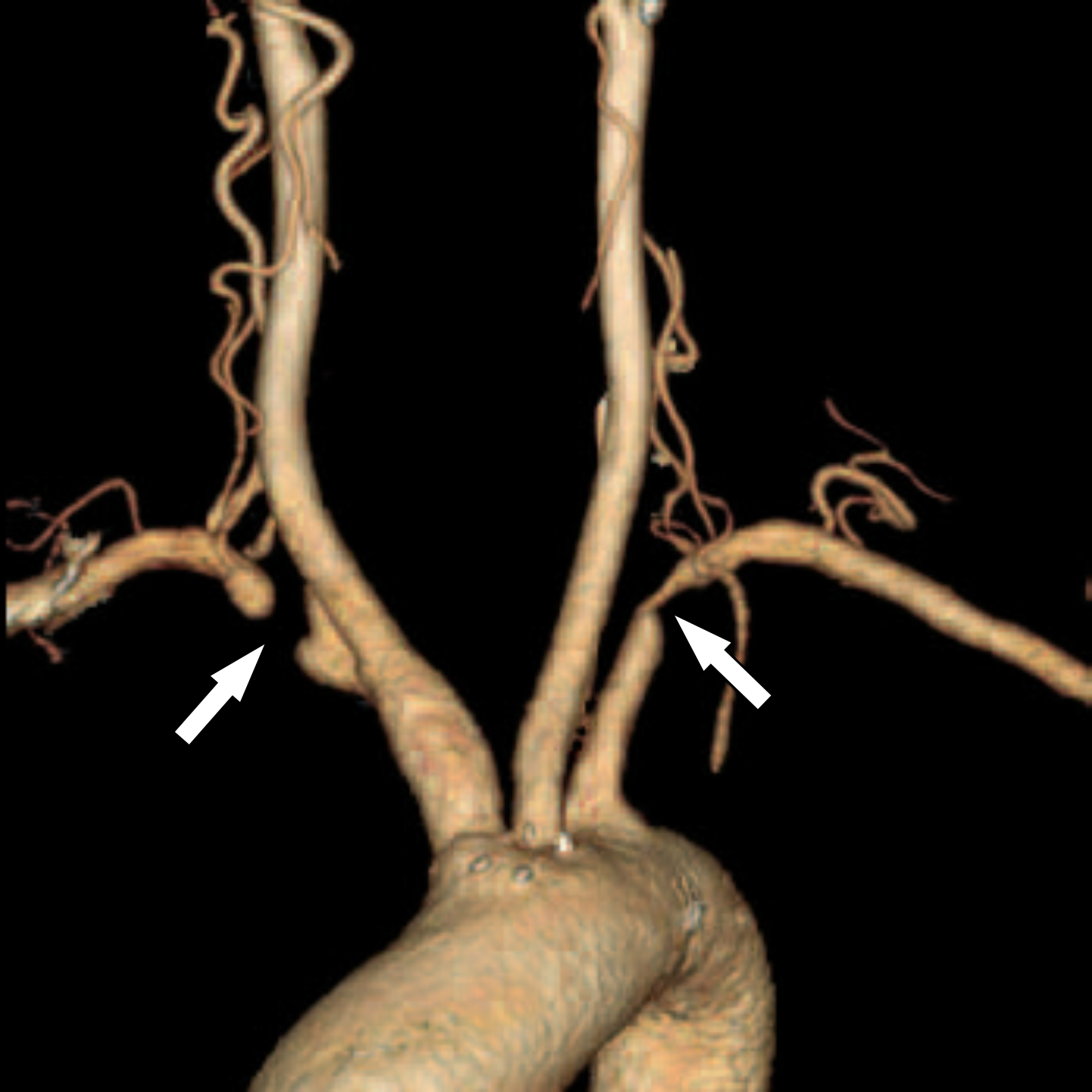


Figure 16 c Figure 16 d

Fig. 16 Compromised delivery paths. A safe endovascular trajectory is needed for safe transportation of the THV to the aortic root. CT is in his respect an essential tool in order to avoid vascular complications and guide to the intervention through the safest possible passage. Potential complications may arise due to luminal narrowing or even chronic iliac artery occlusion with extensive collaterals (a) and pronounced vascular tortuosity and kinking (arrows in b). In this last case, there is an additional short dissection in the left external iliac artery (arrowhead) due to a previously performed conventional coronary angiography. For these patients, the preferred transfemoral access approach is therefore not possible, and other options have to be considered. Nevertheless, other access paths may also pose significant challenges, like bilateral subclavian artery narrowing and occlusion (arrows in c), and the presence of post-infarct thrombus and wall calcification in the left ventricular apex (asterisk in d), making a transapical approach with a balloon-expandable valve impossible. Therefore, vascular access examination must include all anatomic possible entry points for a full assessment of the different options.
